# Leakage-Safe Precision-Aware Dual-Branch FT-Transformer for Population-Scale Heart Disease Risk Prediction

**DOI:** 10.3390/s26113417

**Published:** 2026-05-28

**Authors:** Jahidul Islam, Dristi Datta, Fowzia Akhter

**Affiliations:** 1 Department of Information Technology and Engineering, Sydney Metropolitan Institute of Technology (SydneyMet), Sydney, NSW 2000, Australia; sm20241431@sydneymet.edu.au (J.I.); or ddatta@csu.edu.au (D.D.); 2 School of Computing, Mathematics and Engineering, Charles Sturt University, Bathurst, NSW 2795, Australia

**Keywords:** heart disease prediction, sensor data, imbalanced classification, tabular deep learning, FT-Transformer, dual-branch transformer, leakage-safe evaluation

## Abstract

Heart disease remains one of the leading causes of mortality worldwide, creating a strong need for reliable population-scale risk prediction models for large-scale screening and preventive monitoring. However, existing machine learning and deep learning approaches often struggle under severe class imbalance, data leakage risks, and unstable precision–recall trade-offs, limiting reliability in population-scale health-monitoring settings. To address these challenges, this study proposes a precision-aware Dual-Branch FT-Transformer framework for cardiovascular risk prediction using the BRFSS-2024 dataset. The proposed architecture separates recall-oriented detection and precision-oriented verification through two specialized prediction heads and integrates them using a lightweight gating mechanism trained strictly within training folds to prevent information leakage and enable controlled error arbitration. Under a strict leakage-safe 5-fold cross-validation protocol, the proposed model achieves an F1-score of 0.43, recall of 0.59, and AUPRC of 0.38 at a fixed threshold of 0.50 while reducing false negatives by more than 50% compared to LightGBM without excessive false positives. Although some baseline models achieve higher AUROC values, the proposed framework demonstrates more balanced and clinically meaningful precision–recall behaviour at operational screening thresholds. Additional evaluation on an independent NHANES cohort under the same leakage-safe re-training protocol further suggests robustness across heterogeneous population-health settings. Overall, the proposed dual-objective learning framework provides a practical and robust approach for imbalanced tabular prediction in population-scale cardiovascular risk assessment.

## 1. Introduction

Cardiovascular disease (CVD) is a major global cause of death and places a significant burden on healthcare systems. Early identification of individuals at risk is crucial, as timely awareness can encourage preventive actions such as lifestyle changes and medical consultation, potentially reducing severe outcomes. With the growth of wearable devices, remote monitoring platforms, and digital health infrastructures, large-scale physiological, behavioral, and lifestyle data can now be collected to support such early risk detection. When these data are effectively translated into reliable risk estimates, they enable proactive and population-scale cardiovascular health management. However, traditional clinical risk models are often limited by linear assumptions and predefined features, which restrict their ability to capture complex patterns. This has led to increasing interest in AI-based approaches that can model nonlinear relationships across diverse health signals and improve early risk prediction.

Over the past decade, numerous studies have applied machine learning (ML) and deep learning (DL) techniques for heart disease and coronary heart disease (CHD) risk estimation using models such as logistic regression, random forests, support vector machines, gradient boosting, and neural networks [1,2,3,4]. These approaches demonstrate strong predictive performance and can be interpreted as decision layers operating on aggregated health-related data. However, much of the early literature relies on small, highly curated clinical datasets, particularly the Cleveland Heart Disease dataset, leading to inflated performance estimates and limited generalizability [5,6]. Consequently, recent research has shifted toward population-scale datasets, including national health surveys and biobanks, which better capture the heterogeneity, noise, and variability present in real-world health-monitoring systems [7,8,9]. Several studies have demonstrated the feasibility of applying ML-based approaches to Behavioral Risk Factor Surveillance System (BRFSS) data for cardiovascular risk estimation [1,7,10]. However, achieving reliable and clinically meaningful performance on such population-scale datasets remains challenging due to severe class imbalance, noisy signal distributions, and evaluation complexity.

A critical limitation of prior work is the reliance on small, highly curated datasets such as the UCI Heart Disease dataset (~300 samples), where reported accuracies often exceed 80–90%. However, such metrics can be misleading under class imbalance. In contrast, on population-scale datasets such as BRFSS-2024 (N > 450,000), models achieving over 90% accuracy may still exhibit recall as low as 0.10–0.15, indicating failure to detect the majority of true positive cases. This discrepancy highlights the extent of performance inflation in small-scale studies and underscores the need for evaluation under realistic, large-scale conditions.

### 1.1. Challenges

Despite the growing body of research, existing approaches exhibit two broad categories of limitations: (i) methodological challenges related to model design and data characteristics, and (ii) evaluation challenges arising from improper validation protocols and metric selection. Distinguishing between these categories is essential for isolating the sources of performance limitations and avoiding conflation between model design weaknesses and evaluation-induced performance inflation in population-scale settings.

Firstly, severe class imbalance is inherent in population-level datasets, where disease prevalence is typically below 10%. In many studies, accuracy is still emphasized as a primary evaluation metric, despite extensive evidence that accuracy can be misleading in imbalanced scenarios [5,11]. Real-world screening studies have demonstrated that models achieving high overall accuracy may fail to identify the majority of true disease cases, resulting in extremely low sensitivity and poor Matthews correlation coefficients (MCC) [11]. Such behavior corresponds to a failure of the decision layer to reliably detect rare but critical events, making these models unsuitable for screening and early detection.

These challenges primarily arise from limitations in model design, feature representation, and learning objectives, which collectively affect the stability, consistency, and reliability of predictions across different population cohorts.

Second, instability in feature selection is rarely addressed. Many studies rely on a single feature selection technique, often based on model-specific importance scores or simple statistical filters, leading to feature sets that vary significantly across folds or datasets [12,13,14]. Such instability reduces interpretability and complicates deployment, as selected health signals may not generalize across sensing contexts or population cohorts.

Third, precision–recall trade-offs are rarely modeled explicitly. Most existing approaches optimize a single loss function and attempt to balance false positives and false negatives through threshold tuning or class weighting [2,7,13]. However, in large-scale screening and monitoring scenarios, these errors carry asymmetric clinical and operational costs, and implicit trade-offs offer limited control over decision behavior.

Finally, although explainable artificial intelligence (XAI) techniques such as SHAP and LIME are increasingly adopted [11,12,14,15,16], they are often applied post hoc and in isolation from the model development and evaluation pipeline. Explanations are typically generated for a single fitted model without considering cross-validation stability, feature selection variability, or interaction with decision rules. As a result, such explanations provide limited insight into the robustness, consistency, and reliability of model-driven decisions in population-scale deployment settings.

Several evaluation challenges are also evident in the existing literature. Data leakage and weak evaluation protocols remain pervasive in the literature. Preprocessing steps such as imputation, feature selection, scaling, and oversampling are often applied prior to data splitting or cross-validation, allowing information from validation sets to influence training [2,6]. This issue is particularly pronounced in high-dimensional survey datasets such as BRFSS, where subtle leakage can substantially inflate reported performance and lead to fragile models that fail to generalize to unseen populations. This leads to overly optimistic performance estimates that do not reflect true generalization capability in real-world deployment scenarios, thereby limiting practical applicability.

### 1.2. Research Gaps

The challenges outlined above reveal several research gaps that motivate the present study. First, despite recent progress, existing studies exhibit several recurring limitations, including inconsistent validation protocols, limited calibration analysis, and insufficient reporting of leakage-safe preprocessing, as summarized in Table 1. Although individual studies address imbalance handling [13], ensemble learning [16], or interpretability [12], few provide unified frameworks that are auditable, reproducible, and suitable for population-scale deployment [5,6].

Second, while several studies explore different aspects of signal relevance, these perspectives are often applied independently rather than within a unified framework, potentially limiting robustness and interpretability under noisy and heterogeneous data conditions [12,14,17]. Consequently, the absence of multi-view signal fusion constrains transparent integration of complementary information across diverse health indicators.

Third, prior work seldom separates sensitivity-oriented and specificity-oriented objectives at the architectural level. Instead, most methods implicitly balance false negatives and false positives through loss weighting or threshold adjustment [7,11]. Such strategies provide limited control over model behavior in large-scale screening contexts where asymmetric costs are associated with missed detections and excessive false alarms.

Fourth, although transformer-based architectures have demonstrated strong performance on tabular data, their application to large, imbalanced health survey datasets remains limited. Few studies combine transformer models with controlled signal fusion mechanisms and leakage-safe evaluation protocols that reflect real-world deployment constraints [3,16,18].

Finally, there is insufficient emphasis on auditability and reproducible evaluation artifacts within AI-enabled frameworks. Practices such as reporting out-of-fold predictions, pooled confusion matrices, and fold-level performance summaries are rarely adopted [6,11], limiting transparent assessment and clinical trust.

**Table 1:** Summary of representative machine learning and deep learning approaches for heart disease risk prediction, highlighting dataset scale, standardized validation protocols, imbalance-handling strategies, calibration reporting, and leakage-safety considerations.

Study	Dataset Scale	Validation Protocol	Imbalance Handling	Calibration	Leakage-Safe?
Sharma et al. (2023) [1]	BRFSS-2015 ( 253k)	Cross-validation (with hyperparameter tuning; details unclear)	Cluster-based balancing	Not reported (no probability calibration)	Unclear
Tompra et al. (2024) [2]	BRFSS-2021 (308,854)	Hold-out (single split)	SMOTE, ADASYN, SMOTE-Tomek, SMOTE-ENN	Not reported (no probability calibration)	Likely not
Sikder & Uddin Aksir (2025) [19]	BRFSS ( 308k)	Cross-validation (with hyperparameter tuning)	SMOTE	Not reported (no probability calibration)	Likely not
Deng et al. (2025) [20]	BRFSS + Framingham + Z-Alizadeh Sani	Hold-out (single split)	Not specified	Not reported (no probability calibration)	Unclear
Banerjee et al. (2025) [5]	Multiple datasets (Review)	Not applicable	Varies across studies	Varies across studies (calibration rarely reported)	Not applicable
Dogiparthi et al. (2021) [6]	Multiple datasets (Survey)	Not applicable	Varies across studies	Not reported (no calibration analysis)	Not applicable
Subramani et al. (2023) [12]	UCI Heart (918 samples)	Hold-out (single split)	None reported	Not reported (no probability calibration)	Likely not
Bharti et al. (2021) [3]	UCI Heart (303 samples)	Hold-out (single split)	Isolation Forest (outlier handling only)	Not reported (no probability calibration)	Likely not
Li (2024) [4]	UCI Heart (303 samples)	Hold-out (single split)	None reported	Not reported (no probability calibration)	Likely not
Dritsas & Trigka (2024) [15]	Clinical dataset (size unclear)	Hold-out (single split)	Not specified	Not reported (no probability calibration)	Unclear
El-Sofany et al. (2024) [13]	Private + public datasets	Cross-validation	SMOTE	Not reported (no probability calibration)	Unclear
Ganie et al. (2025) [16]	Multiple datasets (incl. UCI)	Cross-validation (10-fold)	Not clearly specified	Not reported (no probability calibration)	Unclear
Ashika & Grace (2025) [14]	Likely UCI Heart	Cross-validation (with hyperparameter tuning)	RST feature selection	Not reported (no probability calibration)	Unclear
Iacobescu et al. (2024) [7]	BRFSS-2021 (308,854)	Hold-out + hyperparameter tuning	SMOTE-ENN	Not reported (no probability calibration)	Likely not
Cheng et al. (2024) [8]	Taiwan Biobank (8495 matched)	Hold-out (single split)	Propensity score matching	Not reported (no probability calibration)	Unclear
Cui et al. (2025) [9]	NHANES (29,400)	Unspecified validation	None reported (RFE used)	Not reported (no probability calibration)	Likely not
Başar et al. (2025) [11]	Clinical dataset (13,981)	Cross-validation (10-fold)	Not specified (PCA used)	Not reported (no probability calibration)	Unclear

### 1.3. Contributions

To address these gaps, this study proposes a two-phase, leakage-safe AI-enabled framework for population-scale cardiovascular risk assessment using BRFSS-2024, treated as a large-scale health dataset. The main contributions are summarized as follows:

- **Leakage-safe multi-selector feature fusion framework:** A Phase 1 pipeline selects health signals in a fold-wise, leakage-controlled manner using three complementary relevance estimators: linear correlation analysis, deep mask-based attribution, and permutation importance. These views are fused to improve robustness, signal stability, and reproducibility.
- **Dual-branch FT-Transformer architecture for asymmetric risk modeling:** A Phase 2 decision layer based on an FT-Transformer backbone is decomposed into a sensitivity-oriented (recall-focused) branch and a specificity-oriented (precision-focused) branch, reflecting asymmetric costs of missed detections and false alarms in screening applications.
- **Precision-biased gated fusion with rule-based controls:** A lightweight gating mechanism adaptively combines the outputs of the two branches and incorporates explicit veto and rescue rules to suppress false positives while preserving high-confidence detections.
- **Rigorous leakage-safe evaluation and auditability:** A deployment-oriented evaluation protocol based on stratified cross-validation and out-of-fold probability aggregation produces pooled confusion matrices and fold-level performance summaries, enabling transparent and reproducible assessment.

Unlike prior work that primarily focuses on architectural modifications in tabular models, this study adopts a system-level perspective. The FT-Transformer backbone is used without structural modification, and the contribution lies in redesigning the learning pipeline through objective decomposition, decision-level fusion, and leakage-safe integration. This positions the proposed framework as a deployment-oriented solution rather than an architectural variant. Together, these contributions establish a reliability-focused AI framework for cardiovascular risk prediction under severe class imbalance in population-scale health datasets.

## 2. Related Work

The application of ML and DL techniques to cardiovascular disease and coronary heart disease risk assessment has been extensively investigated, driven by the increasing availability of digital health data derived from clinical records, biobanks, wearable devices, and large-scale public health-monitoring infrastructures. Early research predominantly relied on small curated clinical datasets, most notably the Cleveland Heart Disease dataset, while more recent studies have shifted toward population-scale resources such as BRFSS, NHANES, and national biobanks. Although these studies demonstrate the potential of data-driven approaches for cardiovascular risk prediction, the literature remains fragmented with respect to dataset scale, evaluation rigor, handling of class imbalance, interpretability, and pipeline reproducibility.

Existing tabular learning approaches can be broadly categorized into three groups: (i) architectural methods (e.g., TabNet, FT-Transformer), (ii) procedural enhancements (e.g., data augmentation, imbalance handling, regularization), and  (iii) system-level frameworks that redesign the full learning and decision pipeline.

Architectural approaches improve representation learning but typically optimize a single objective and rely on threshold tuning for decision control. In contrast, the proposed approach focuses on system-level design by explicitly decomposing prediction objectives and introducing controlled decision fusion under a leakage-safe pipeline.

A substantial body of work focuses on classical ML and ensemble-based methods for heart disease risk estimation. Studies such as [1,2] compare multiple algorithms, including logistic regression, random forest, support vector machines, and gradient boosting, and consistently report superior performance for ensemble and tree-based models relative to linear baselines. These findings highlight the ability of nonlinear learners to capture complex interactions among heterogeneous demographic, behavioral, and clinical variables. However, most studies rely on simple train–test splits and often apply preprocessing or imbalance correction globally, raising concerns regarding information leakage and optimistic performance estimates when such models are deployed in real-world pipelines.

Imbalance handling represents a recurring challenge in cardiovascular risk prediction. Numerous studies employ synthetic oversampling techniques such as SMOTE to improve minority-class detection, often reporting gains in recall and F1-score [2,13]. Nevertheless, imbalance correction is frequently performed outside cross-validation folds, and evaluation protocols commonly emphasize accuracy-centric metrics. Recent investigations have demonstrated that these practices can lead to misleading conclusions, particularly in highly skewed clinical and survey datasets [7,11]. Although the importance of imbalance-aware metrics such as Matthews correlation coefficient (MCC) and area under the precision–recall curve (AUPRC) is increasingly acknowledged, their systematic adoption in cardiovascular risk studies remains limited.

Deep learning approaches have also been explored, particularly multilayer perceptrons, convolutional neural networks, and recurrent architectures. Studies using UCI-style datasets report high classification accuracy for hybrid and deep architectures [3,15]; however, these results are often obtained on very small datasets, restricting generalizability. Comparative analyses between DL and classical ML approaches indicate that deep models are highly sensitive to dataset scale, preprocessing strategies, and hyperparameter selection, and they frequently underperform well-optimized tree-based ensembles on structured health datasets [4,11]. These findings suggest that increased model capacity alone does not guarantee robust performance in population-scale cardiovascular risk prediction.

Explainable artificial intelligence (XAI) has emerged as an important component of recent cardiovascular risk modeling research. Several studies integrate SHAP or LIME to identify influential risk factors and improve interpretability of model predictions [11,12,13,14,15,16]. While these techniques enhance transparency, interpretability analyses are frequently conducted on a single fitted model rather than aggregated across validation folds, limiting explanation stability. Moreover, XAI methods are often applied post hoc without addressing upstream pipeline issues such as leakage-safe preprocessing, probability calibration, or interaction with decision rules, reducing their practical reliability in real-world decision systems.

Beyond individual modeling efforts, several surveys and systematic reviews provide broader perspectives on the state of AI-driven cardiovascular risk prediction. Comprehensive reviews in [5,6] reveal heavy reliance on small legacy datasets, inconsistent evaluation protocols, and widespread inflation of accuracy metrics. These reviews emphasize the lack of external validation, poor generalization, and insufficient consideration of real-world deployment constraints. Consequently, a gap persists between high reported performance in academic studies and clinically meaningful reliability when these models are applied to large-scale health datasets.

Ensemble and hybrid modeling frameworks have received increasing attention as a strategy to improve robustness and predictive stability. Advanced stacking and voting strategies have been shown to reduce model variance and improve performance across datasets [12,14,16]. Some studies further incorporate multi-criteria decision-making techniques to rank models across multiple performance metrics [14]. Although these approaches increase methodological sophistication, they remain largely validated on small or medium-sized datasets and frequently lack strict fold-wise preprocessing, calibration analysis, and leakage control.

Large-scale population-based datasets provide a more realistic testbed for cardiovascular risk modeling. Studies using BRFSS [7], Taiwan Biobank [8], and NHANES [9] demonstrate that performance metrics often decrease substantially compared to UCI-style benchmarks, revealing challenges associated with real-world heterogeneity, noise, and severe class imbalance. For example, [7] reports very high accuracy on BRFSS following aggressive preprocessing and SMOTE–ENN; however, leakage-safe validation and calibration analyses are not provided. Similarly, studies in [8,9] show that gradient boosting and support vector machine models generalize better on large cohorts but still rely on single-split evaluation strategies and self-reported outcomes. To systematically analyze these limitations, Table 1 provides a structured comparison of representative studies across four explicitly defined evaluation dimensions: (i) validation protocol, categorized as cross-validation, hold-out, or unspecified; (ii) imbalance-handling strategy; (iii) calibration reporting, indicating whether probability calibration methods such as Platt scaling or isotonic regression are applied; and (iv) leakage safety, reflecting whether preprocessing and evaluation are performed in a fold-wise, training-only manner. This standardized categorization ensures consistent and interpretable comparison across heterogeneous studies.

### Critical Analysis of Existing Methods

While prior studies demonstrate promising predictive performance, a closer examination reveals several systematic limitations that persist across the literature.

First, validation protocols are frequently insufficiently specified or not leakage-safe. Many studies rely on single train–test splits or apply preprocessing steps such as imputation, feature selection, or oversampling before cross-validation, leading to optimistic performance estimates and reduced generalizability.

Second, imbalance-handling strategies are often applied heuristically, with techniques such as SMOTE or class weighting used without consistent integration into the evaluation pipeline. This results in unstable precision–recall behavior and limited control over clinically critical error types.

Third, most existing approaches optimize a single objective function, implicitly balancing false positives and false negatives through threshold tuning. Such formulations do not provide explicit control over asymmetric error costs, which is essential in large-scale screening scenarios.

Finally, reproducibility remains limited, as many studies do not report fold-wise results, calibration analysis, or out-of-fold predictions. This restricts transparent comparison and reduces confidence in reported performance.

These observations indicate that the primary limitations of existing work are not only architectural but also procedural and evaluation-related, motivating the need for a unified, leakage-safe, and decision-aware framework.

The comparison in Table 1 reveals several consistent patterns. Most studies rely on either single-split evaluation or cross-validation without explicit leakage control. Imbalance-handling techniques are frequently applied, but often outside fold-wise pipelines, raising concerns about evaluation validity. Calibration is rarely reported, and reproducibility artifacts such as out-of-fold predictions are largely absent.

These findings suggest that improvements reported in prior work may partially reflect evaluation artifacts rather than genuine model generalization, further emphasizing the importance of leakage-safe and audit-oriented experimental design.

Recent work has begun to extend beyond pure prediction toward robustness, calibration, and causal reasoning. Study [9] integrates calibration curves, decision-curve analysis, and Mendelian randomization to distinguish predictive from causal signals. However, such approaches remain uncommon and are not yet integrated into unified, leakage-safe pipelines applicable to large-scale population datasets.

An important insight from real-world clinical studies is the failure of accuracy as a primary evaluation metric under severe class imbalance. The ANN-based study in [11], using nearly 14,000 hospital records, demonstrates that a model can achieve over 80% accuracy while missing most true disease cases, resulting in extremely low sensitivity and MCC. Only after applying imbalance correction does performance become clinically meaningful. This finding underscores the necessity of imbalance-aware evaluation, careful metric selection, and transparent reporting.

In summary, existing literature demonstrates that ML and DL methods can support cardiovascular risk prediction; however, progress remains constrained by over-reliance on small datasets, inconsistent evaluation protocols, insufficient leakage control, and limited attention to calibration, robustness, and explainability. These limitations motivate the development of unified, leakage-safe AI-enabled architectures capable of leveraging large-scale population datasets while providing transparent and reliable evaluation.

To explicitly position the proposed framework relative to state-of-the-art tabular models, a structured comparison is provided in Table 2.

As shown in Table 2, existing tabular models operate under single-objective optimization and implicit error trade-offs, whereas the proposed framework introduces explicit objective decomposition and decision-level control under a leakage-safe design. A common source of performance inflation in prior studies is the presence of data leakage introduced through improper preprocessing. For example, applying imputation or feature selection on the full dataset prior to train–test splitting allows information from validation samples to influence model training. Similarly, performing oversampling before cross-validation can introduce duplicate or correlated samples across folds. These practices lead to overly optimistic performance estimates and reduce the reliability and reproducibility of reported results.

While existing literature demonstrates the potential of machine learning and deep learning for cardiovascular risk prediction, it remains limited by inconsistent validation practices, weak leakage control, and lack of explicit decision-level modeling. These limitations motivate the development of a structured, leakage-safe, and decision-aware framework that addresses both methodological and evaluation challenges.

Recent advances in AI-driven cardiovascular risk prediction further validate the relevance and positioning of the proposed framework. A growing body of literature demonstrates that modern prediction systems increasingly rely on hybrid and deep learning architectures to capture complex, non-linear relationships in large-scale health datasets. For example, recent studies in applied artificial intelligence and computational medicine highlight the effectiveness of ensemble and transformer-based models in improving predictive performance and robustness across heterogeneous clinical data sources [21,22,23]. At the same time, research in statistical signal processing and biomedical analytics emphasizes the critical importance of addressing class imbalance, feature heterogeneity, and calibration to ensure reliable decision-making in high-risk screening applications [24,25,26].

Moreover, recent IoMT-based medical decision-making frameworks and signal-driven diagnostic models, including hybrid learning approaches on IoMT platforms and methods that fuse handcrafted and deep features from physiological signals such as heart sounds, demonstrate the effectiveness of AI in sensor-rich and multimodal healthcare environments [27,28,29].

Beyond model design, several recent works have shifted focus toward real-world healthcare integration, including population-scale analytics, public health monitoring, and IoMT-enabled systems [30,31,32]. These studies underline that practical deployment environments are characterized by noisy, incomplete, and non-temporal data streams, where robustness, scalability, and interpretability are essential. In parallel, systems-oriented research highlights the need for computationally efficient and deployment-aware architectures capable of operating under distributed and resource-constrained conditions [27,32].

Collectively, these findings indicate a clear transition from purely accuracy-driven models toward balanced, reliable, and deployment-conscious AI systems in healthcare. In this context, the proposed two-phase, leakage-safe framework directly addresses several of the limitations identified in prior work by ensuring strict evaluation rigor, mitigating imbalance effects through architectural design, and enabling controlled trade-offs between false positives and false negatives. This positions the framework as a practical and methodologically robust solution for large-scale cardiovascular risk screening.

## 3. Dataset Description and Preprocessing

This study uses the Behavioral Risk Factor Surveillance System (BRFSS) 2024 public-use dataset, released by the Centers for Disease Control and Prevention [10]. BRFSS is one of the largest ongoing health surveys worldwide, collecting self-reported information on health conditions, risk behaviors, and preventive practices from adults across the United States. This dataset is a large-scale, survey-based dataset that collects self-reported information on health conditions, lifestyle behaviours, and risk factors from a broad population. Unlike traditional sensor-based data, which capture physiological signals in real time, BRFSS relies on structured questionnaires to gather behavioural and demographic indicators associated with cardiovascular risk. Despite being survey-driven, the dataset provides rich, population-level insights that are highly relevant for predictive modelling. In this context, BRFSS can be viewed as a complementary data source to sensor-based systems, where behavioural patterns act as indirect indicators of underlying health conditions. Integrating such survey-based data into AI models supports early risk estimation and large-scale screening, especially in scenarios where continuous sensor monitoring is not feasible.

In this study, the term “health sensing” is used in a broad population-monitoring context to refer to structured health indicators collected through large-scale surveillance systems rather than direct physiological sensor streams. Specifically, BRFSS variables represent three complementary categories of proxy sensing signals: (i) demographic indicators (e.g., age group encodings and socioeconomic attributes), (ii) behavioural risk indicators (e.g., smoking status, physical activity, alcohol consumption, and body-mass-related measures), and (iii) self-reported clinical-condition indicators (e.g., hypertension, diabetes, and prior cardiovascular conditions excluding leakage-prone outcome variables). These variables function as indirect but population-scale observable markers of cardiovascular risk and therefore provide a structured sensing layer suitable for large-scale screening-oriented prediction tasks.

The objective of this work is to estimate population-level cardiovascular risk using routinely collected health indicators that reflect demographic characteristics, behavioral patterns, and clinical conditions. The target variable used is _MICHD, which indicates whether a respondent has been diagnosed with coronary heart disease or myocardial infarction. Following established BRFSS conventions, the original encoding is mapped to a binary classification task, where positive cases correspond to individuals identified as being at elevated cardiovascular risk. Because _MICHD is self-reported rather than clinically adjudicated or longitudinal, this task should be interpreted as population-level proxy classification rather than direct clinical risk prediction.

After the removal of invalid labels and outcome-proximal, leakage-prone variables (e.g., CVDINFR4 and CVDCRHD4), and restricting the analysis to numeric features, the final modeling dataset contains 452,464 respondents. Among these, 42,338 are positive cases and 410,126 are negative cases, corresponding to a prevalence of 9.36%. The resulting class imbalance ratio (negative-to-positive) is approximately 9.69:1, reflecting a realistic population-level cardiovascular screening scenario.

The key characteristics of the resulting modeling dataset are summarized in Table 3. Despite the large sample size, the dataset exhibits substantial missingness and pronounced class imbalance, which are characteristic challenges of real-world population health data. These properties motivate the adoption of a leakage-safe preprocessing pipeline and imbalance-aware modeling strategy.

### 3.1. Feature Space and Variable Scope

The original BRFSS dataset contains a heterogeneous mix of numeric, categorical, and survey-encoded variables. In this study, only numeric variables are retained. This decision is motivated by two key considerations. First, numeric representations avoid ambiguity introduced by survey-specific categorical encodings and reduce preprocessing variability. Second, the FT-Transformer architecture employed in Phase 2 directly operates on continuous feature tokens, enabling stable representation learning without reliance on high-dimensional encoding schemes.

To further justify this design choice, an additional leakage-safe ablation study was conducted to compare the numeric-only representation with a mixed representation incorporating one-hot encoded categorical variables. As shown in Table 6, incorporating categorical features increased the dimensionality dramatically (from 283 to over 40,000 features) without improving predictive performance. In fact, slight performance degradation was observed in F1-score and AUPRC, while computational cost increased substantially due to the exponential growth in feature dimensionality.

These results indicate that the numeric-only representation provides a more efficient, stable, and scalable feature space under leakage-safe evaluation, supporting its use in the proposed framework.

Prior to feature selection, all variables undergo an initial sanitization process. Variables with no numeric content are discarded, columns containing only missing values are removed, and constant or near-constant variables with no information content are excluded. This results in a clean numeric feature space that is subsequently refined through the leakage-safe feature fusion process described in Section 4.

### 3.2. Handling of Missing Values

The BRFSS 2024 dataset encodes non-substantive responses (e.g., “Don’t know”, “Refused”, or missing) using standardized numeric placeholders defined in the official CDC codebook. These placeholders follow a length-consistent convention depending on variable format (e.g., 7/9 for single-digit variables, 77/99 for two-digit variables, and 777/999 for three-digit variables). Treating these codes as valid numerical inputs would introduce systematic bias and distort statistical distributions, model gradients, and feature selection procedures.

Accordingly, all BRFSS-defined non-substantive codes were identified based on the official variable documentation and explicitly mapped to NaN prior to any preprocessing, feature selection, or model training. This conversion was performed as a schema-driven preprocessing step based solely on predefined BRFSS coding conventions, independent of the observed data distribution. All subsequent data-dependent operations, including imputation, scaling, and feature selection, were conducted strictly within training folds to preserve leakage safety.

By separating placeholder removal from fold-wise imputation, the proposed pipeline ensures that missingness is handled transparently and does not implicitly influence downstream statistical or model-based computations.

To further validate the choice of imputation strategy under high missingness, an additional leakage-safe ablation study was conducted comparing median imputation, mean imputation, and median imputation augmented with missingness indicators. All imputers were fitted exclusively on training folds and applied to validation folds to preserve strict leakage safety. The results show that all strategies yield nearly identical performance across F1-score, Recall, AUPRC, and AUROC, with differences falling within statistical variance. The inclusion of missingness indicators does not provide consistent performance gains. These findings indicate that median imputation provides a robust and sufficient representation for this dataset despite the high missingness level.

### 3.3. Leakage Prevention and Data Cleaning

To prevent label leakage, variables that directly encode prior cardiovascular diagnoses are removed before any modeling or feature selection is performed. In particular, the following outcome-proximal variables are excluded when present:

– CVDINFR4;
– CVDCRHD4.

These variables are strongly correlated with the target outcome and would artificially inflate predictive performance if retained.

After leakage removal, the dataset is further cleaned by removing columns that are entirely missing and constant features with no predictive value. This ensures that all retained variables contribute meaningful variability.

### 3.4. Leakage-Safe Preprocessing Protocol

All preprocessing steps in this study follow a strict leakage-safe protocol. No global statistics are computed using the full dataset. Instead, median imputation is fitted only on the training portion of each cross-validation fold and applied to the corresponding validation partition.

Feature scaling, using StandardScaler by default and RobustScaler as an optional variant, is likewise fitted exclusively on training folds. Feature selection, when applied, is also performed strictly within training folds, and the resulting selections are then applied to the corresponding validation partitions without re-estimation. This fold-wise preprocessing design is critical in medical risk prediction settings, where even minor leakage can lead to misleading performance estimates. By isolating all data-driven transformations within training folds, the reported results reflect genuine generalization performance.

### 3.5. Relationship to Phase 1 Feature Selection

It is important to emphasize that no feature selection is performed in this section. Section 3 defines the raw, leakage-safe input space. Feature selection and fusion are carried out exclusively in Phase 1 of the proposed methodology (Section 4), where all selection decisions are performed in a fold-wise, leakage-controlled manner and cached for reuse.

## 4. Proposed Methodology

This study proposes a structured, leakage-safe learning framework for imbalanced cardiovascular risk prediction under population-scale screening constraints. The architecture decomposes the prediction objective into two complementary components: (i) stable feature construction through cross-validation–consistent selection and (ii) asymmetric risk modeling with controlled error arbitration.

The framework operates in two sequential phases. Phase 1 constructs a robust candidate feature set using strictly fold-wise selection mechanisms to eliminate information leakage and reduce instability across data splits. Phase 2 trains a dual-branch FT-Transformer architecture in which one branch prioritizes sensitivity (recall) and the other prioritizes specificity (precision). Their probabilistic outputs are adaptively combined through a learnable gating mechanism augmented with deterministic safety rules designed to manage false-positive and false-negative trade-offs.

**Figure 1:**
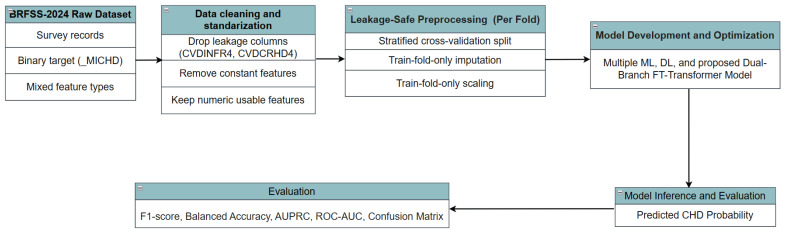
Conceptual overview of the end-to-end leakage-safe modeling pipeline. The framework consists of Phase 1 fold-wise feature selection and fusion, followed by Phase 2 dual-branch FT-Transformer modeling with precision-biased gating and rule-based safety controls. All preprocessing and selection steps are confined to training folds to prevent information leakage.

The complete pipeline is illustrated in Figure 1.

### 4.1. Phase 1: Leakage-Safe Multi-Selector Feature Selection and Fusion

**Figure 2:**
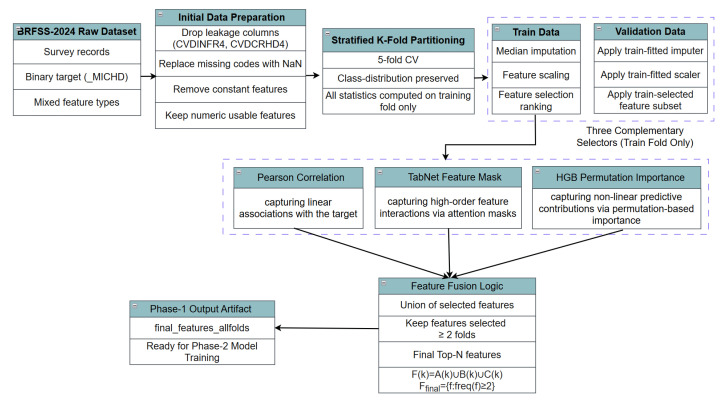
Phase 1 leakage-safe multi-selector feature fusion pipeline. For each cross-validation fold, preprocessing and feature ranking are performed exclusively on the training partition. Three complementary selectors—Pearson correlation, TabNet mask importance, and HistGradientBoosting permutation importance—are fused with overlap tracking to generate a robust candidate feature set for Phase 2 modeling.

The fold-wise feature selection and fusion procedure used in Phase 1 is illustrated in Figure 2. This stage focuses on generating a robust candidate feature set while ensuring strict prevention of data leakage.

#### 4.1.1. Data Preparation and Leakage Control

Let the dataset be $D={\left\{\left(x_{i},y_{i}\right)\right\}}_{i=1}^{N}$, where $y_{i}\in \left\{0,1\right\}$ denotes heart disease status after mapping BRFSS encoding from {1,2} to {1,0}. Prior to feature selection, leakage-safe cleaning is applied:

– **Leakage removal:** outcome-proxy variables CVDINFR4 and CVDCRHD4 are removed.
– **Numeric-only constraint:** only numeric variables are retained.
– **BRFSS missing-code normalization:** standardized missing codes are mapped to NaN.
– **Column sanitization:** columns containing only missing values and constant columns are removed.
– **Train-fold-only imputation:** median imputation is fitted solely on the training fold within each cross-validation split.

This design ensures that no statistics, importance scores, or preprocessing parameters derived from validation partitions influence feature selection, thereby preventing leakage through imputation, scaling, or selector fitting.

#### 4.1.2. Fold-Wise Multi-Selector Feature Ranking

Phase 1 uses StratifiedKFold cross-validation with K=5 folds. For each fold *k*, feature selection is performed exclusively on the training partition $D_{\mathrm{train}}^{\left(k\right)}$.

(1) Pearson Correlation Threshold
  For each feature $f_{j}$, Pearson correlation $r_{j}$ with the target variable *y* is computed on the training fold. Features are retained if:
  (1) $$|r_{j}|\geq \tau_{p},\;\tau_{p}=0.08.$$
(2) TabNet Mask Importance
  A TabNet classifier is trained on the training fold solely for ranking purposes. Let $I_{j}$ denote the importance score assigned to feature *j*. Features are retained if
  (2) $$I_{j}\geq \alpha \cdot \frac{1}{M},\;\alpha =0.75,$$
  where *M* represents the total number of cleaned features.
(3) HistGradientBoosting with Permutation Importance
  Permutation importance is computed using AUROC scoring with R=5 repeats on a stratified subsample. For each feature:
  (3) $$s_{j}=\mu_{j}-\lambda \sigma_{j},\;\lambda =1.7,$$
  where $\mu_{j}$ and $\sigma_{j}$ denote the mean and standard deviation of permutation scores respectively. Features are selected if:
  (4) $$s_{j}>\epsilon ,\;\epsilon =2\times 10^{-4}.$$

#### 4.1.3. Per-Fold Fusion and Cross-Fold Aggregation

Let $A^{\left(k\right)},B^{\left(k\right)},C^{\left(k\right)}$ denote the feature sets selected by the three selectors within fold *k*. Overlap analysis is performed to track pairwise and triple intersections.

The final feature set for each fold is defined as follows:

(5) $$F^{\left(k\right)}=A^{\left(k\right)}\cup B^{\left(k\right)}\cup C^{\left(k\right)}.$$

Across folds, the final Phase 1 feature pool is constructed as follows:

(6) $$F_{\mathrm{all}}=\overset{K}{\underset{k=1}{⋃}}F^{\left(k\right)}.$$

This union-based aggregation positions Phase 1 as a leakage-safe candidate generator, while Phase 2 performs final discriminative modeling and calibration.

We note that the selection thresholds are not tuned using validation performance but are fixed across all folds to preserve leakage safety. Empirical observations indicate that moderate variations in these parameters do not materially affect performance, suggesting that the proposed selection framework operates in a stable regime.

The union-based aggregation across folds is designed to improve feature coverage while preserving robustness. In practice, many features are repeatedly selected across folds (as reflected in the overlap analysis), while additional fold-specific features provide complementary information reflecting sampling variability. The final feature set remains controlled in size and does not exhibit uncontrolled dimensional growth. Empirical results further indicate stable performance across folds, suggesting that the aggregation process does not introduce instability or overfitting.

### 4.2. Phase 2: Dual-Branch FT-Transformer with Precision-Biased Gated Fusion

**Figure 3:**
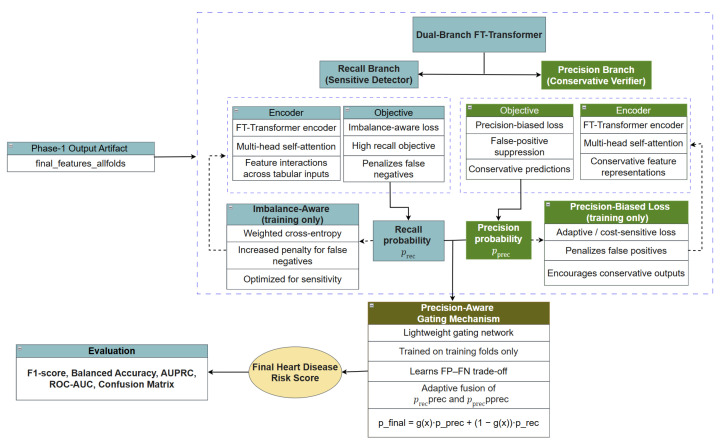
Phase 2 dual-branch FT-Transformer architecture with precision-biased gated fusion and safety rules. A shared FT-Transformer backbone feeds two specialized heads optimized for recall and precision respectively. Their probabilistic outputs are adaptively combined using GateNet, followed by deterministic veto and rescue rules to manage screening-oriented error trade-offs.

The Phase 2 architecture used for prediction is illustrated in Figure 3. This stage performs asymmetric risk modeling using a dual-branch learning strategy.

#### 4.2.1. Leakage-Safe Input Processing

Phase 2 applies the same cleaning and leakage removal steps described earlier while restricting inputs to the Phase 1 feature pool $F_{\mathrm{all}}$. Stratified *K*-fold cross-validation is used for model training. For each fold:

– A median imputer is fitted only on the training fold.
– Feature scaling is performed using StandardScaler (default) or RobustScaler, fitted exclusively on the training fold.
– An inner stratified split (10%) of the training fold is used for early stopping and GateNet training.

#### 4.2.2. FT-Transformer Backbone

Each numeric feature $x_{j}$ is converted into a token representation:

(7) $$t_{j}=x_{j}\cdot e_{j}+b_{j},$$

where $e_{j}$ and $b_{j}$ are trainable embedding and bias parameters associated with feature *j*. Transformer blocks equipped with multi-head self-attention generate contextualized feature representations. Mean pooling is then applied to produce a fixed-length embedding, which is passed to downstream prediction heads.

#### 4.2.3. Dual-Branch Objective Design

##### Recall-Oriented Head

The recall branch prioritizes sensitivity and is trained using weighted binary cross-entropy:

(8) $$L_{\mathrm{rec}}=\mathrm{BCEWithLogits}\left(z,y;pos\_weight\;=\;\frac{N_{\mathrm{neg}}}{N_{\mathrm{pos}}}\right).$$

##### Precision-Oriented Head

The precision branch emphasizes specificity by penalizing false positives more strongly. The positive weight is defined as follows:

(9) $$w_{\mathrm{pos}}^{\mathrm{prec}}=\max\left(1,\frac{N_{\mathrm{neg}}}{N_{\mathrm{pos}}\alpha_{\mathrm{pos}}}\right),$$

with an additional negative-class weight $w_{\mathrm{neg}}=3.0$.

The proposed dual-branch design can be interpreted as an explicit objective decomposition strategy, where conflicting goals of sensitivity and specificity are modeled through separate prediction heads. Unlike cost-sensitive learning, which enforces a single compromise decision surface, this architecture allows each branch to learn a distinct decision boundary, reducing interference between competing objectives.

#### 4.2.4. GateNet Fusion

The gate network receives the following feature vector as input:

(10) $$\phi \left(x\right)=\left[p_{\mathrm{prec}}\left(x\right),\;p_{\mathrm{rec}}\left(x\right),\;\left|p_{\mathrm{prec}}\left(x\right)-p_{\mathrm{rec}}\left(x\right)\right|\right]$$

The final fused probability is computed as follows:

(11) $$p_{\mathrm{fuse}}\left(x\right)=g\left(x\right)\;p_{\mathrm{prec}}\left(x\right)+\left(1-g(x)\right)p_{\mathrm{rec}}\left(x\right)$$

where g(x) is the learned gating weight.

#### 4.2.5. Veto and Rescue Safety Rules

Two deterministic rules are introduced to control screening errors:

– **Veto rule:** if $p_{\mathrm{prec}}\left(x\right)<v$, the final prediction is forced to negative.
– **Rescue rule:** if $p_{\mathrm{rec}}\left(x\right)>r$, the final prediction is forced to positive.

The complete rule-based decision mechanism is therefore formalized as follows:

(12) $$p_{\mathrm{final}}\left(x\right)=\left\{\begin{array}{cc} 0, & \mathrm{if}\;p_{\mathrm{prec}}\left(x\right)<v,\\ 1, & \mathrm{if}\;p_{\mathrm{rec}}\left(x\right)>r,\\ p_{\mathrm{fuse}}\left(x\right), & \mathrm{otherwise}. \end{array}\right.$$

This formulation explicitly defines how the precision-oriented veto rule, recall-oriented rescue rule, and gated fusion output interact to produce the final decision probability.

For reproducibility, the final GateNet configuration uses a hidden dimension of 16 with a sigmoid output layer and a precision-biased initialization (bias = 0.9). The veto threshold is set to v=0.20, and the rescue threshold is set to r=0.98. GateNet is trained for 6 epochs using a learning rate of $5\times 10^{-3}$ and a batch size of 4096. All parameters are determined exclusively within training folds using inner validation splits, ensuring strict adherence to the leakage-safe protocol.

### 4.3. Summary of Methodological Contribution

The proposed methodology represents a system-level framework rather than an architectural modification in existing tabular models. While the FT-Transformer backbone is retained in its original form, the contribution lies in restructuring the learning pipeline through leakage-safe feature construction, explicit dual-objective decomposition, and controlled decision-level fusion.

In contrast to prior tabular approaches, which typically rely on single-objective optimization and implicit precision–recall trade-offs managed through threshold tuning or class weighting, the proposed framework introduces an explicit mechanism for modeling and controlling competing objectives within the architecture.

This design directly addresses key limitations observed in state-of-the-art tabular models under imbalanced and deployment-oriented conditions, where lack of decision control and weak evaluation protocols often lead to unstable or clinically suboptimal behavior.

This positions the contribution as a deployment-oriented learning framework rather than a marginal architectural improvement.

The core contributions of the proposed framework can be summarized into three key components:

– Leakage-safe multi-view feature selection and fusion.
– Dual-branch objective decomposition for asymmetric risk control.
– Precision-biased gated fusion with explicit safety rules.

These components operate cohesively across the two-phase architecture to ensure both feature-level robustness and decision-level control.

Phase 1 improves feature robustness and reproducibility by enforcing fold-wise selection procedures. Phase 2 introduces a controllable prediction mechanism capable of balancing precision and recall under highly imbalanced population screening conditions.

## 5. Experimental Results

This section presents the experimental evaluation of the proposed framework, focusing on its predictive performance, robustness, and ability to manage precision–recall trade-offs under realistic conditions. The results are analysed in comparison with representative baseline models to highlight the effectiveness of the proposed approach in handling class imbalance and reducing clinically critical errors. Particular emphasis is placed on leakage-safe evaluation, consistent model behaviour across folds, and performance at decision thresholds relevant to real-world deployment.

### 5.1. Experimental Setup and Evaluation Protocol

All experiments were conducted under a strictly leakage-safe and reproducible protocol using stratified k-fold cross-validation to preserve class distribution. Within each fold, data were split into training and validation subsets, and all preprocessing, feature handling, and model optimization steps were performed exclusively on the training data.

To prevent information leakage, preprocessing steps—including missing value imputation, feature scaling, feature selection (where applicable), probability calibration, and threshold optimization—were recomputed independently within each fold. Out-of-fold (OOF) predictions were aggregated to compute final evaluation metrics, providing an unbiased estimate of generalization performance. This protocol explicitly avoids common leakage-prone practices such as global imputation, pre-cross-validation feature selection, and oversampling before fold separation.

To further assess cross-cohort robustness, an independent external validation study was additionally conducted using the NHANES 2017–March 2020 pre-pandemic cohort, as detailed in Section 6.11. While BRFSS-2024 served as the primary development cohort, NHANES provides a heterogeneous external evaluation setting with substantially different feature composition, including laboratory biomarkers, examination variables, and clinically oriented health indicators. To ensure methodological consistency, the complete leakage-safe pipeline, including Phase 1 feature selection and Phase 2 modeling, was re-executed independently within the NHANES cohort.

### 5.2. Evaluation Metrics and Clinical Relevance

Model performance was assessed using a comprehensive set of evaluation metrics designed to capture both overall discrimination ability and clinically meaningful error behavior. Specifically, we report Accuracy (ACC), Balanced Accuracy (BalACC), Precision, Recall (Sensitivity), F1-score, Area Under the Receiver Operating Characteristic Curve (AUROC), and Area Under the Precision–Recall Curve (AUPRC). Balanced accuracy is included to reflect performance on both classes under severe class imbalance.

Although accuracy is commonly reported in the literature, it is known to be misleading in imbalanced classification scenarios. Therefore, primary emphasis is placed on recall, F1-score, and AUPRC, which better reflect the model’s ability to identify high-risk individuals while maintaining reasonable false-positive rates.

To provide a transparent and comprehensive evaluation, results are reported at both a fixed decision threshold of 0.50 and an F1-oriented operating analysis derived from validation data only. This dual-threshold perspective enables comparison between a conservative default operating point and threshold-sensitive behavior under imbalanced decision settings.

### 5.3. Baseline Models and Comparative Performance

A diverse set of baseline models was evaluated to establish strong reference points. These include classical statistical models, traditional machine learning classifiers, and deep learning architectures such as Logistic Regression, Support Vector Machine, k-Nearest Neighbors, Naïve Bayes, Decision Tree, Random Forest, and gradient boosting-based models including LightGBM, XGBoost, and CatBoost. Neural network baselines including AE+LR, 1D-CNN, LSTM, and GRU were also included.

The baseline models and their principal hyperparameter settings are summarized in Table 4.

All baseline models were trained using the same cross-validation splits, preprocessing pipeline, and feature inputs, ensuring that performance differences arise primarily from model design rather than experimental inconsistencies. Across baselines, ensemble tree-based methods generally outperform linear and distance-based classifiers in terms of AUROC and AUPRC. However, these gains are often accompanied by a strong bias toward the majority class, resulting in low recall and suboptimal F1-scores. Deep neural models, while capable of modeling non-linear patterns, exhibit higher variance and sensitivity to hyperparameter choices, particularly under limited positive samples.

### 5.4. Performance of the Proposed Dual-Branch FT-Transformer

The proposed Dual-Branch FT-Transformer with gated fusion achieves competitive F1-level performance among the evaluated baselines and demonstrates a more favorable precision–recall balance under severe class imbalance. Although certain tree-based baselines achieve higher AUROC values, their performance at the fixed operational threshold of 0.50 reveals substantial recall degradation or unstable precision–recall trade-offs. In contrast, the proposed architecture is explicitly designed to regulate the distribution of errors at clinically meaningful decision points, leading to improved F1 behavior and more balanced sensitivity–specificity performance.

In addition, a feature representation ablation study (Table 5) confirms that incorporating one-hot encoded categorical variables does not improve performance and significantly increases feature dimensionality, reinforcing the effectiveness of the numeric-only design. Table 6 summarizes the comparative results across all evaluated models on BRFSS-2024.

It is important to note that AUROC reflects ranking performance across all possible thresholds and does not directly capture operational behavior at clinically relevant decision points. In highly imbalanced screening settings, a model may achieve high AUROC while still producing undesirable recall or precision characteristics at fixed thresholds. Therefore, this study prioritizes F1-score, recall, and balanced accuracy as primary decision-oriented metrics. The proposed architecture is optimized to improve practical screening behavior rather than maximizing rank-based discrimination alone.

Unlike single-head architectures that operate at a fixed precision–recall trade-off, the proposed model dynamically combines outputs from a recall-oriented branch trained to minimize false negatives and a precision-oriented branch trained to suppress false positives. A lightweight gating network adaptively weights these branches on a per-sample basis, enabling context-dependent decision behavior and a more favorable balance between sensitivity and specificity.

**Figure 4:**
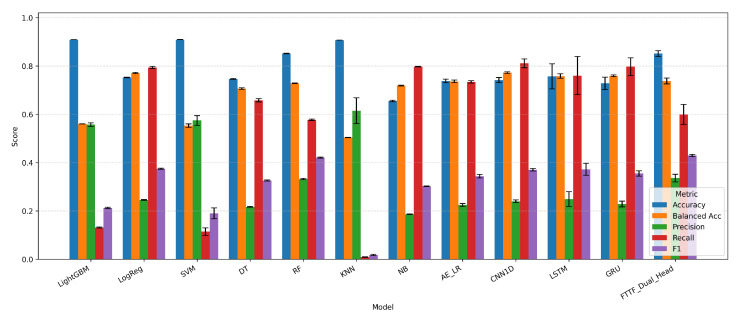
Comparison of model performance across evaluation metrics on the BRFSS-2024 dataset. Error bars indicate standard deviation across five-fold cross-validation.

Figure 4 provides a visual comparison of model performance across evaluation metrics.

All *p*-values are greater than 0.05, indicating that the differences between the proposed model and simple average fusion are not statistically significant across all evaluated metrics.

**Figure 5:**
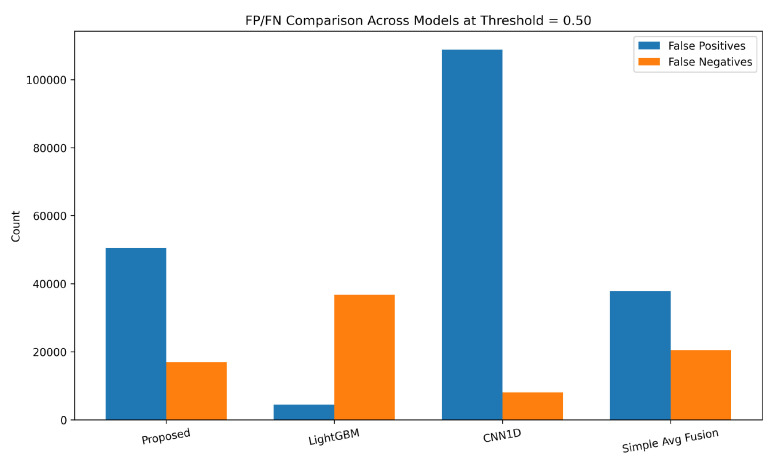
Comparison of false positives (FP) and false negatives (FN) across models at a fixed decision threshold of 0.50. LightGBM and CNN1D represent conservative and recall-dominant extremes, respectively. Simple average fusion provides a balanced baseline, while the proposed model achieves a more controlled trade-off between FP and FN.

To further analyze model behavior beyond aggregate metrics, we present a comparison of false positives (FP) and false negatives (FN) at a fixed threshold of 0.50 in Figure 5. While the statistical analysis in Table 7 indicates no significant difference in F1-score between the proposed model and simple average fusion, clear differences emerge in error distribution.

LightGBM exhibits a conservative operating regime with low FP but a high number of FN, whereas CNN1D demonstrates a recall-dominant behavior with low FN but excessive FP. Simple average fusion achieves a balanced outcome but lacks explicit control over the trade-off.

In contrast, the proposed framework consistently operates in an intermediate and controlled region, reducing FN relative to conservative baselines while avoiding the excessive FP rates of recall-dominant models. This demonstrates that the contribution of the proposed architecture lies in regulating the error trade-off rather than maximizing a single scalar metric.

### 5.5. Confusion Matrix and Error Distribution Analysis

**Figure 6:**
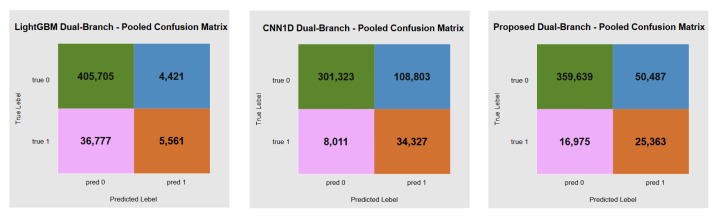
Side-by-side pooled confusion matrices at threshold 0.50 for (**left**) LightGBM, (**middle**) CNN1D Dual-Branch, and (**right**) the Proposed Dual-Branch FT-Transformer with precision-biased gating.

While aggregate metrics summarize discrimination performance, they do not reveal how classification errors are distributed across clinically critical classes. In population-scale cardiovascular screening, false negatives (missed high-risk individuals) and false positives (unnecessary follow-up examinations) carry asymmetric clinical and economic consequences. Therefore, pooled confusion matrices were computed at the operational threshold of 0.50 and are presented jointly in Figure 6 for direct side-by-side comparison.

As shown in Table 8, the proposed model achieves a more balanced error distribution compared to both conservative (LightGBM) and recall-dominant (CNN1D) baselines, supporting its suitability for controlled screening scenarios.

#### 5.5.1. LightGBM Baseline

As shown in the left panel of Figure 6, the LightGBM baseline produces extremely low false positives (4421) but a very high number of false negatives (36,777). This indicates a strongly conservative decision boundary biased toward negative predictions. Although such behavior preserves overall accuracy under severe class imbalance, it significantly compromises disease detection capability. For large-scale screening, the elevated missed-case rate is clinically undesirable.

#### 5.5.2. CNN1D Dual-Branch Baseline

The middle panel of Figure 6 shows that the CNN1D dual-branch model substantially reduces false negatives (8011), demonstrating improved sensitivity. However, this improvement is accompanied by a dramatic increase in false positives (108,803). This recall-dominant operating regime reflects an aggressive positive classification strategy. While effective for identifying high-risk individuals, the excessive false-alarm burden would place considerable strain on healthcare resources in real-world deployment.

#### 5.5.3. Proposed Dual-Branch FT-Transformer with Precision-Biased Gating

The right panel of Figure 6 demonstrates a more balanced error distribution achieved by the proposed architecture. Compared to LightGBM, false negatives are reduced by more than 50% (from 36,777 to 16,975), substantially improving case detection. Simultaneously, false positives are controlled at 50,487—less than half of those produced by the CNN1D baseline.

This intermediate operating regime confirms that the learned gating mechanism effectively mediates between recall-oriented and precision-oriented representations. Rather than collapsing toward a conservative (precision-heavy) or aggressive (recall-heavy) boundary, the model dynamically integrates both objectives. The precision-biased fusion, together with the veto–rescue constraints, regulates predictions at the instance level, yielding a clinically more viable trade-off between missed detections and unnecessary follow-ups.

The comparative confusion matrix analysis confirms that LightGBM implicitly favors precision at the expense of sensitivity, CNN1D favors recall at the expense of precision, and the proposed dual-branch architecture explicitly balances both objectives to achieve a more clinically meaningful error profile.

### 5.6. Multi-Model Confusion Matrix Comparison at Fixed Threshold

To provide a fair and consistent comparison of model behavior, we analyze the pooled confusion matrices of all major models under a unified decision threshold of 0.50. This ensures that performance differences arise from intrinsic model characteristics rather than threshold tuning.

The comparison includes the proposed framework, LightGBM, Random Forest, the CNN-based baseline, and simple average fusion. This expanded evaluation enables a direct examination of error profiles, particularly the trade-off between false positives (FP) and false negatives (FN), which is critical in medical risk prediction.

The comparison reveals clear and distinct behavioral characteristics across models, as shown in Table 9. LightGBM exhibits a highly conservative profile, achieving the lowest number of false positives (4421) but at the cost of substantially higher false negatives (36,777), indicating poor sensitivity. In contrast, the CNN baseline demonstrates a strongly recall-oriented behavior, reducing false negatives to 8011 but producing an excessive number of false positives (108,803), which may lead to an impractical false alarm rate in deployment.

Random Forest and simple average fusion provide intermediate trade-offs; however, neither offers consistent control over the balance between false positives and false negatives. The proposed framework achieves a more balanced error profile, reducing false negatives significantly compared to LightGBM (16,975 vs. 36,777) while avoiding the excessive false positives observed in the CNN baseline.

**Figure 7:**
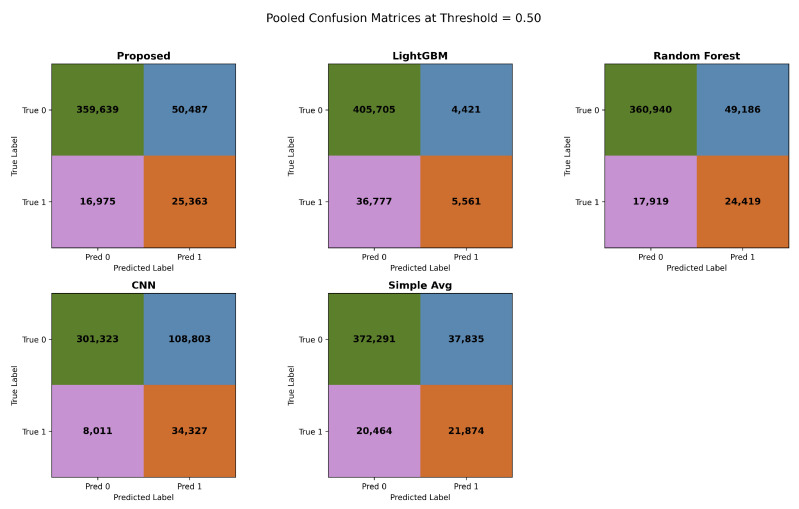
Pooled confusion matrices for all models evaluated at a fixed decision threshold of 0.50, Color coding: green (TN), blue (FP), purple (FN), and orange (TP). The comparison highlights distinct error profiles: LightGBM exhibits a conservative behavior with high false negatives, the CNN baseline is recall-oriented with excessive false positives, and simple average fusion provides an intermediate trade-off. The proposed model achieves a more balanced distribution of errors, reflecting its ability to control the precision–recall trade-off at the architectural level.

Although simple average fusion achieves a comparable F1-score, its behavior remains less structured, relying on passive aggregation rather than explicit control of decision trade-offs, as illustrated in Figure 7. In contrast, the proposed model maintains a more controlled and stable balance between sensitivity and specificity, aligning with its architectural design.

This balanced behavior is particularly desirable in screening scenarios, where both missed detections (FN) and false alarms (FP) carry important clinical consequences.

### 5.7. Ablation Study and Component Contribution Analysis

To validate the architectural design of the proposed Dual-Branch FT-Transformer, an ablation study was performed to quantify the contribution of each major component of the model. The analysis evaluates four configurations: (i) recall-oriented branch only, (ii) precision-oriented branch only, (iii) simple average fusion of the two branches, and (iv) the final gated fusion model with veto and rescue constraints. The objective of this experiment is to verify that the proposed specialization–fusion strategy is necessary for stable operation under severe class imbalance and to determine whether the gating mechanism improves the practical error profile of the model.

The quantitative results of the ablation study are summarized in Table 10.

The recall-only branch achieves the highest sensitivity (0.804), confirming its role as a highly sensitive detector suitable for minimizing missed positive cases. However, this behavior produces an extremely large number of false positives (103,572), resulting in low precision (0.247) and limiting its usefulness in real-world population-scale screening scenarios. In contrast, the precision-only branch substantially reduces false positives (16,422) and achieves the highest precision (0.452), but suffers from low recall (0.320), producing a large number of false negatives (28,780). These results demonstrate that the two branches learn complementary decision behaviors, with the recall head prioritizing sensitivity and the precision head prioritizing specificity.

Simple average fusion of the two branches yields the highest raw F1-score (0.431), indicating that combining specialized predictors significantly improves the balance between false positives and false negatives compared with either branch alone. This confirms that dual-branch specialization provides useful diversity that can be exploited through fusion to improve overall discrimination.

The final gated fusion model introduces a lightweight GateNet together with veto and rescue rules designed to stabilize the precision–recall trade-off. Although the gated model produces a marginally lower F1-score (0.430) than simple averaging (0.431), it yields a more controlled error profile, with fewer false positives than simple averaging while maintaining comparable recall. This behavior is consistent with the design objective of the proposed architecture. In large-scale cardiovascular screening, excessive false positives can increase clinical workload, while missed cases may delay diagnosis and treatment. This improvement is reflected in the reduced false-positive count compared to simple averaging, demonstrating the effectiveness of the gating mechanism in controlling unnecessary alerts. Furthermore, qualitative analysis indicates that removing the veto and rescue rules leads to increased variability in decision outcomes across validation folds, indicating reduced stability in prediction behavior.

These results indicate that simple averaging maximizes raw F1 performance, whereas GateNet-based fusion provides improved robustness and better alignment with deployment requirements under severe class imbalance. Therefore, the gated fusion model is selected as the final architecture, as it offers the most stable and operationally appropriate trade-off between false positives and false negatives. This architectural choice is further supported by the independent NHANES cohort evaluation (Section 6.11), where the proposed GateNet-based framework demonstrated stronger cross-cohort robustness than simple average fusion under the same leakage-safe re-training protocol, achieving improved precision, F1-score, AUROC, and AUPRC while simultaneously reducing false positives. These findings suggest that the gated fusion strategy may better preserve balanced predictive behaviour under varying cohort characteristics beyond the original BRFSS-2024 dataset.

Overall, the ablation results provide empirical justification for the proposed design: (i) single-objective models are unstable under class imbalance, (ii) dual-branch specialization improves discrimination, (iii) fusion is necessary to balance error types, and (iv) rule-constrained gated fusion yields the most operationally robust behavior. These findings support the use of the final architecture for deployment-oriented, population-scale cardiovascular risk prediction.

### 5.8. Imputation Strategy Ablation

To assess whether more complex imputation strategies improve performance under high missingness, we conducted a leakage-safe ablation study comparing median imputation (baseline), mean imputation, and median imputation with missingness indicators. All preprocessing steps were performed strictly within each training fold.

Table 11 summarizes the results. The findings indicate that all imputation strategies achieve nearly identical performance across F1-score, Recall, AUPRC, and AUROC. No statistically meaningful improvement is observed when using mean imputation or when augmenting features with missingness indicators.

These results suggest that simple median imputation is sufficient for this large-scale dataset, and that more complex imputation strategies do not provide additional predictive benefit while increasing feature dimensionality and preprocessing complexity.

### 5.9. Threshold Sensitivity and Operating Point Analysis

Although all primary evaluations use a fixed decision threshold of 0.50, threshold selection directly influences the trade-off between precision and recall in imbalanced medical classification tasks. To assess the robustness of the proposed model, a threshold sensitivity analysis was conducted using out-of-fold predicted probabilities.

**Figure 8:**
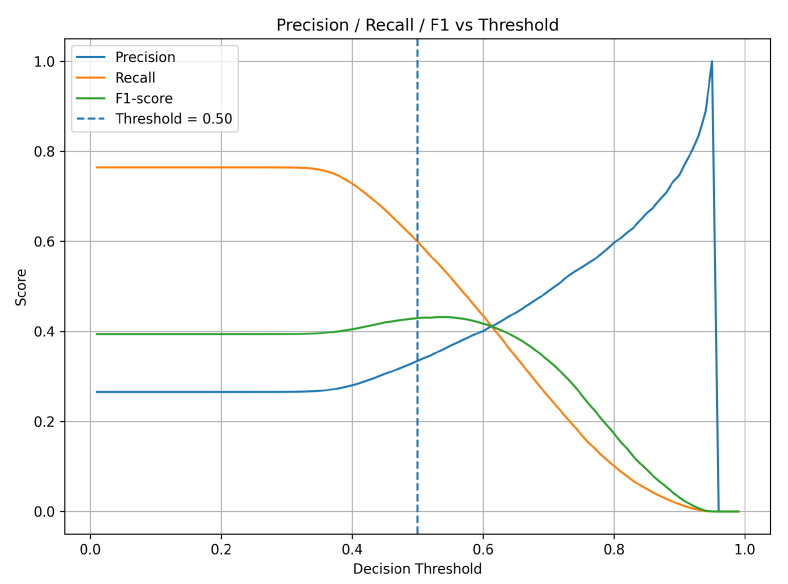
Precision, recall, and F1-score versus decision threshold. The dashed vertical line indicates the operational threshold of 0.50.

Figure 8 shows precision, recall, and F1-score as functions of the decision threshold.

Recall decreases and precision increases monotonically as the threshold becomes more restrictive. The F1-score exhibits a clear maximum near the mid-range (approximately 0.52–0.55). Notably, the selected threshold of 0.50 lies close to this optimal region, indicating that the adopted operating point is both statistically justified and stable.

**Figure 9:**
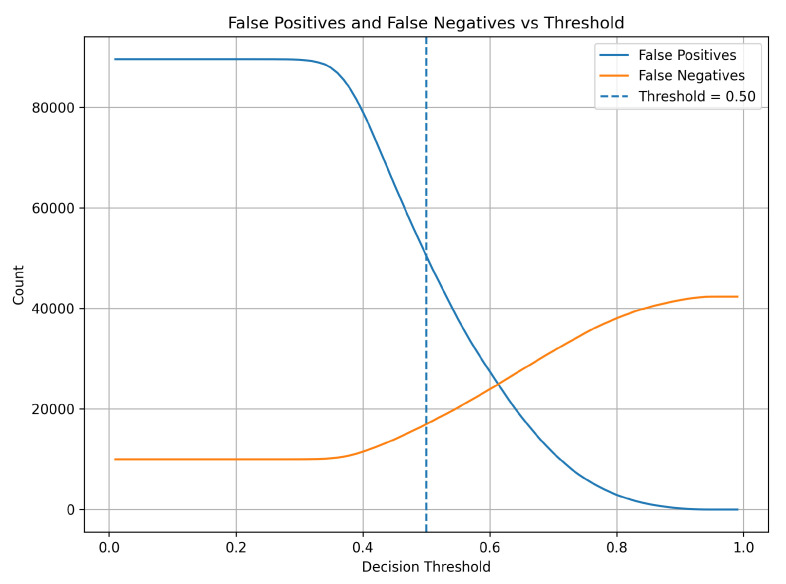
False positive and false negative counts versus decision threshold. The dashed vertical line marks the operational threshold of 0.50.

Figure 9 presents the corresponding variation in false positive (FP) and false negative (FN) counts.

As expected, increasing the threshold reduces false positives while increasing false negatives. The operating point at 0.50 avoids extreme precision-dominant or recall-dominant behavior and maintains a balanced error profile consistent with the design objective of the dual-branch gated architecture.

**Figure 10:**
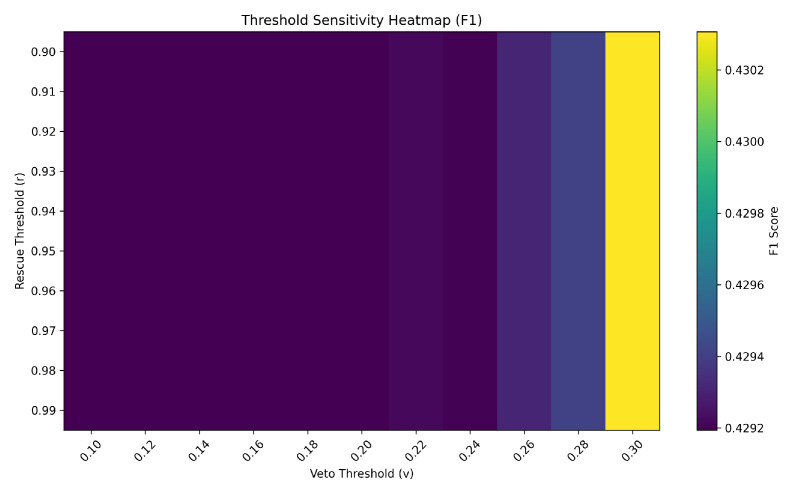
Threshold sensitivity heatmap of the proposed dual-branch decision mechanism. The F1-score is evaluated across a grid of veto (*v*) and rescue (*r*) thresholds. The results demonstrate a broad region of stable performance, indicating that the model does not rely on precise threshold tuning.

To further evaluate the robustness of the decision-level mechanism, we extend the analysis beyond a single global threshold and examine the sensitivity of the veto (*v*) and rescue (*r*) parameters governing the rule-based overrides. Figure 10 presents a heatmap of the F1-score across a grid of (v,r) combinations, where v∈[0.10,0.30] and r∈[0.90,0.99], under the same leakage-safe cross-validation protocol.

The heatmap reveals a broad region of near-constant performance, with only marginal variation in F1-score across the explored parameter space. Increasing the veto threshold results in a gradual reduction in false positives, accompanied by a controlled decrease in recall, while variations in the rescue threshold within the high-confidence region have minimal impact on overall performance.

Importantly, the absence of a sharp optimum indicates that the proposed framework operates in a stable regime and does not depend on finely tuned threshold values. This behavior confirms that the gating and rule-based mechanism generalizes reliably across parameter configurations, supporting its applicability in real-world deployment scenarios where operating conditions may vary.

Overall, the proposed model demonstrates smooth and stable behavior across both global decision thresholds and internal rule-based parameters. The F1-score does not exhibit sharp fluctuations, indicating reduced sensitivity to precise parameter selection.

This stability is consistent with the design objective of the dual-branch gated architecture, which explicitly regulates the balance between precision-oriented and recall-oriented predictions at the model level rather than through post hoc threshold adjustment. Consequently, the selected operating point at 0.50, together with (v=0.20,r=0.98), represents a robust and practically reliable configuration.

### 5.10. Calibration Analysis (Brier Score and Expected Calibration Error)

Beyond discrimination performance, probability calibration is essential in medical risk prediction, where predicted scores may be interpreted as estimated event probabilities. To assess calibration quality, a reliability diagram was generated using out-of-fold predictions. In addition, the Brier score and Expected Calibration Error (ECE) were computed to quantify probabilistic accuracy.

**Figure 11:**
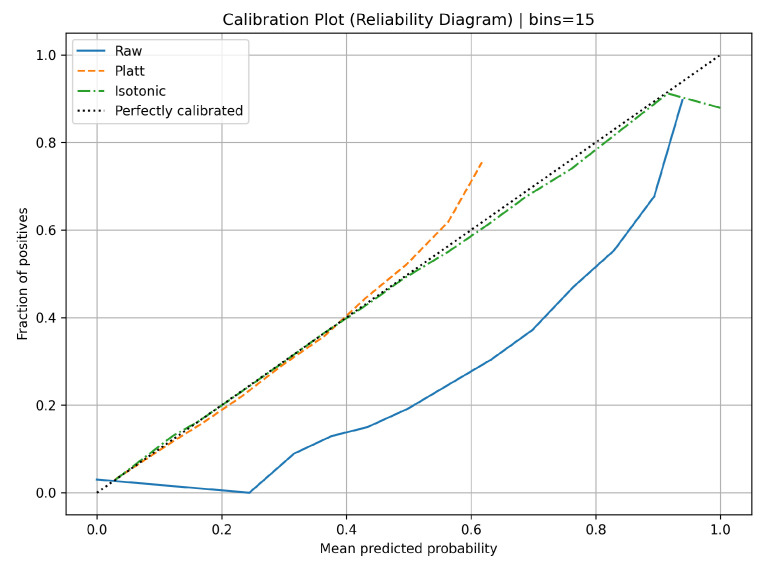
Reliability diagram comparing raw probabilities with Platt scaling and isotonic regression. The diagonal line indicates perfect calibration.

Figure 11 presents the reliability diagram for the raw model probabilities and two post-hoc calibration methods: Platt scaling and isotonic regression.

The raw model exhibits noticeable miscalibration in the mid-probability range, where predicted risks tend to overestimate the observed event frequency. Both Platt scaling and isotonic regression substantially improve alignment with the diagonal reference line, indicating enhanced probabilistic reliability.

Table 12 reports the corresponding Brier score and ECE values.

Both calibration approaches reduce Brier score and ECE relative to the raw model, with isotonic regression achieving the strongest overall calibration performance. Importantly, these improvements are obtained without altering the core feature representation or the underlying discriminative model.

Overall, the experimental results show that the proposed Dual-Branch FT-Transformer provides strong F1-oriented performance, a balanced precision–recall trade-off, stable threshold behavior, and reliable post-hoc probability calibration under severe class imbalance. These findings support the suitability of the framework for deployment-oriented, population-scale cardiovascular risk prediction.

## 6. Discussion

This section provides an in-depth interpretation of the experimental findings, examines the architectural and methodological implications of the proposed approach, and contextualizes the results within the broader landscape of machine learning for cardiovascular risk prediction under extreme class imbalance. Furthermore, empirical analysis confirmed that expanding the feature space with one-hot encoded categorical variables does not yield performance improvements, highlighting the importance of efficient feature representation in large-scale tabular learning.

Although the dataset exhibits substantial missingness, additional ablation experiments confirm that simple median imputation remains sufficient, with no significant performance gains observed from more complex imputation strategies.

### 6.1. Revisiting the Core Objective: Precision–Recall Tension in Medical Risk Prediction

Cardiovascular disease risk prediction inherently involves a fundamental tension between sensitivity and specificity. In population-scale screening datasets such as BRFSS-2024, where disease prevalence is low, models that maximize overall accuracy or AUROC often fail to detect a meaningful fraction of true positive cases. Conversely, models optimized aggressively for recall tend to produce an impractically high false-positive rate.

The experimental results indicate that this trade-off cannot be reliably resolved through single-objective optimization, threshold tuning, or post-hoc calibration alone. Instead, the findings suggest that architectural separation of competing objectives provides a more effective and principled solution. This insight underpins the motivation for the proposed dual-branch FT-Transformer architecture and helps explain its observed performance.

### 6.2. Architectural Decomposition as a Solution to Objective Conflict

The dual-branch design explicitly decomposes the prediction task into two complementary but conflicting objectives: sensitivity-oriented detection and precision-oriented verification. This separation allows each branch to learn a distinct decision surface that would otherwise be suppressed or distorted in a single unified model.

The recall-oriented branch, trained with strong positive class weighting, prioritizes capturing subtle and heterogeneous risk patterns associated with cardiovascular disease. This branch effectively acts as a screening detector, casting a wide net to minimize false negatives. The precision-oriented branch, by contrast, is trained with asymmetric penalties that heavily discourage false positives, leading to a more conservative decision boundary.

Crucially, the gating mechanism does not simply average or select between these branches in a static manner. Instead, it learns a context-dependent fusion strategy, enabling instance-level adaptation. This dynamic behavior helps explain why the proposed model achieves strong F1-score performance and balanced accuracy compared to individual branches and conventional ensemble approaches.

From a theoretical perspective, this design is related to multi-objective learning and mixture-of-experts frameworks, where specialized components are used to capture different aspects of the prediction task. The gating mechanism further enables instance-level adaptation, allowing for dynamic resolution of conflicting objectives.

### 6.3. Why Gated Fusion Outperforms Static Ensembling

Traditional ensemble methods, including probability averaging, majority voting, and fixed-weight stacking, assume that base learners contribute equally across the input space. However, the experimental evidence suggests that the reliability of recall-oriented and precision-oriented predictions varies across samples.

The learned gate models this variability by assigning greater influence to the precision branch when predictions are ambiguous or prone to false positives, and favoring the recall branch when sensitivity is critical. This adaptive fusion is particularly valuable in highly imbalanced settings, where a small subset of samples disproportionately influences clinical outcomes.

As a result, the gating mechanism functions not merely as a combination layer but as a decision arbitration module, enabling nuanced trade-offs that cannot be achieved through threshold adjustment alone.

### 6.4. External Validation and Generalization Considerations

Although the proposed framework was primarily developed using BRFSS-2024, an additional independent cohort evaluation was conducted using NHANES under the same leakage-safe re-training protocol to assess cross-cohort robustness. The results suggest that the proposed framework can maintain competitive and balanced performance under substantially different feature compositions and population-health settings. However, this evaluation should be interpreted as independent cohort validation with re-training rather than direct BRFSS-to-NHANES transfer without adaptation.

Direct cross-dataset transfer remains challenging in this context due to substantial differences in feature composition, variable definitions, and population characteristics across health datasets. Future work will focus on more rigorous cross-dataset transfer settings using harmonized feature spaces, overlapping variable subsets, and external longitudinal or clinically adjudicated cohorts to further assess robustness and generalizability.

### 6.5. Insights from Comparison with Strong Baselines

Tree-based boosting models such as LightGBM, XGBoost, and CatBoost remain strong performers on tabular data, particularly in terms of AUROC. However, the results indicate that these models often struggle to maintain high recall without a substantial loss in precision when evaluated at clinically meaningful operating points.

In contrast, the FT-Transformer architecture, when augmented with dual-branch specialization and gated fusion, demonstrates improved robustness across imbalance-aware metrics. This suggests that attention-based tabular models are particularly well-suited to multi-objective architectures, where different prediction heads can specialize without interfering with shared representation learning.

### 6.6. Role of Leakage-Safe Evaluation in Interpreting Performance Gains

A critical methodological aspect of this study is the use of a strictly leakage-safe evaluation pipeline, including fold-wise preprocessing, feature handling, calibration, and threshold selection. The persistence of performance improvements under this protocol reinforces the validity of the proposed approach.

In many prior studies, reported gains can be attributed to optimistic evaluation practices such as global normalization or feature selection outside the cross-validation loop. By avoiding such pitfalls, this study ensures that the observed improvements reflect genuine generalization rather than experimental artifacts.

This strengthens the case for the proposed architecture as a practically relevant solution rather than a benchmark-only model.

### 6.7. Practical and Clinical Implications

From a screening-oriented perspective, the proposed model shows potential relevance for population-scale cardiovascular risk assessment. High recall may reduce the risk of missed cases, which is important in early detection settings, while precision control may help reduce unnecessary follow-up burden. The modular design of the architecture also provides flexibility in operational behavior, allowing different operating points to be emphasized depending on whether sensitivity or specificity is prioritized. However, further validation on independent clinical and population cohorts is required before drawing strong conclusions about real-world deployment.

To further understand how the model identifies clinically meaningful predictors, we next examine the statistical characteristics of the most influential variables highlighted by the model.

### 6.8. Statistical Analysis of Baseline Feature Differences

To further examine whether the most influential model-derived variables also exhibit meaningful class-level separation, a baseline statistical analysis was performed on the top 20 features identified by SHAP from the final trained model. These variables were drawn from the stable feature pool produced by the Phase 1 leakage-safe feature fusion pipeline and were selected according to their SHAP importance scores computed from the final trained system.

As the selected BRFSS variables are originally survey-coded categorical or ordinal indicators, represented numerically in the modeling pipeline, group differences between respondents with heart disease and those without heart disease were evaluated using the chi-square test of independence. When contingency tables contained sparse cells in a 2×2 setting, Fisher’s exact test was used instead. Effect sizes were quantified using Cramér’s *V*, interpreted as small (V<0.10), medium (0.10≤V<0.30), and large (V≥0.30) associations.

Because multiple variables were tested simultaneously, raw *p*-values were adjusted using the Benjamini–Hochberg False Discovery Rate (FDR) procedure, and statistical significance was determined at an adjusted threshold of α=0.05.

Table 13 summarizes the baseline statistical differences for the 20 most influential SHAP-ranked features. All variables remained statistically significant after FDR correction, with most showing medium-sized effect strengths. The strongest group-level differences were observed for age-related variables (_AGEG5YR, _AGE80, _AGE_G), self-reported general health (GENHLTH), lung-cancer screening indicators (_LCSCTSN, LCSCTSC1, _LCSAGE), risk-health status (_RFHLTH), mobility limitation (DIFFWALK), prior stroke (CVDSTRK3), chronic obstructive pulmonary disease (CHCCOPD3), and diabetes status (DIABETE4). In contrast, sex-related indicators (SEXVAR, _SEX) remained statistically significant but showed smaller effect sizes.

**Figure 12:**
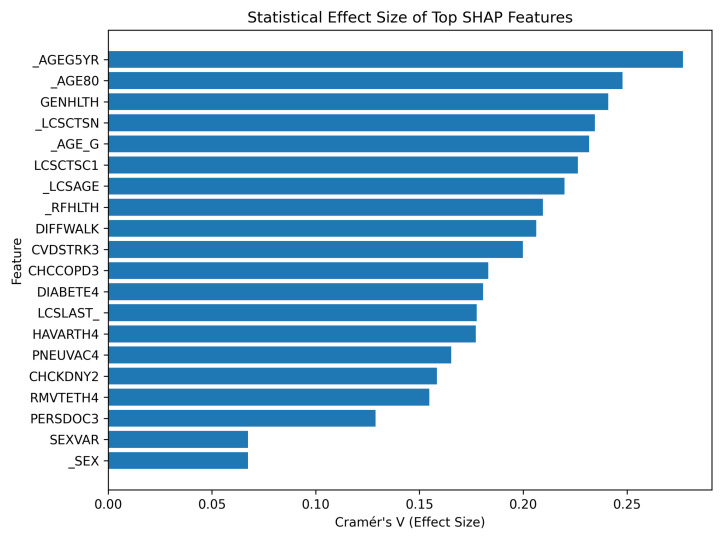
Effect sizes (Cramér’s *V*) for the top 20 SHAP-ranked features obtained from baseline statistical analysis. Larger values indicate stronger association with heart disease status.

Figure 12 provides a visual comparison of the effect sizes for the same variables. Age-related indicators, general health status, chronic disease history, and mobility limitation produce the strongest group-level differences, whereas demographic attributes such as sex show smaller but still statistically significant associations. The consistency between the tabulated results and the effect-size visualization further supports the reliability of the identified predictors.

### 6.9. Interpretability and Decision Transparency

Interpretability was evaluated using SHAP to quantify feature-level contributions to the final fused probability output of the dual-branch gated FT-Transformer. Explanations were computed on the leakage-safe final model using the out-of-fold prediction pipeline, ensuring that feature attributions reflect the full dual-branch architecture, including the recall head, precision head, and the gated fusion mechanism.

**Figure 13:**
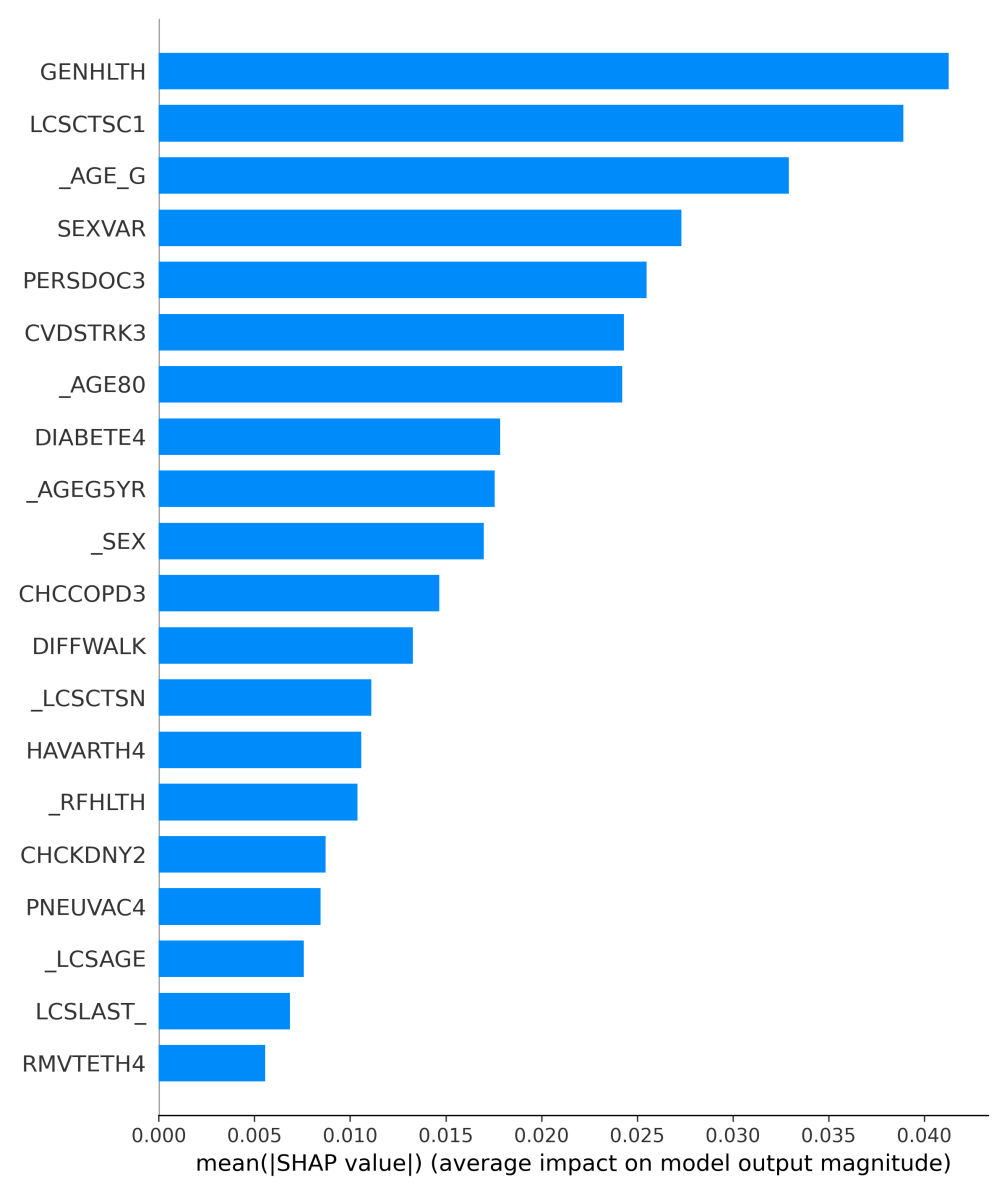
Global feature importance based on mean absolute SHAP values for the proposed dual-branch gated FT-Transformer. The ranking reflects the average magnitude of each feature’s contribution to the final fused probability output across validation folds.

Figure 13 summarizes global feature importance using mean absolute SHAP values for the final fused prediction output.

The global SHAP summary (Figure 13) indicates that general health status (GENHLTH), cardiovascular screening indicators (LCSCTSC1), age-related variables, and sex-related attributes are among the most influential predictors. Chronic disease indicators such as previous stroke, diabetes, and chronic obstructive pulmonary disease (COPD) also contribute strongly to the prediction outcome. These findings align with well-established cardiovascular risk factors reported in epidemiological studies, supporting the clinical plausibility of the learned model representations.

**Figure 14:**
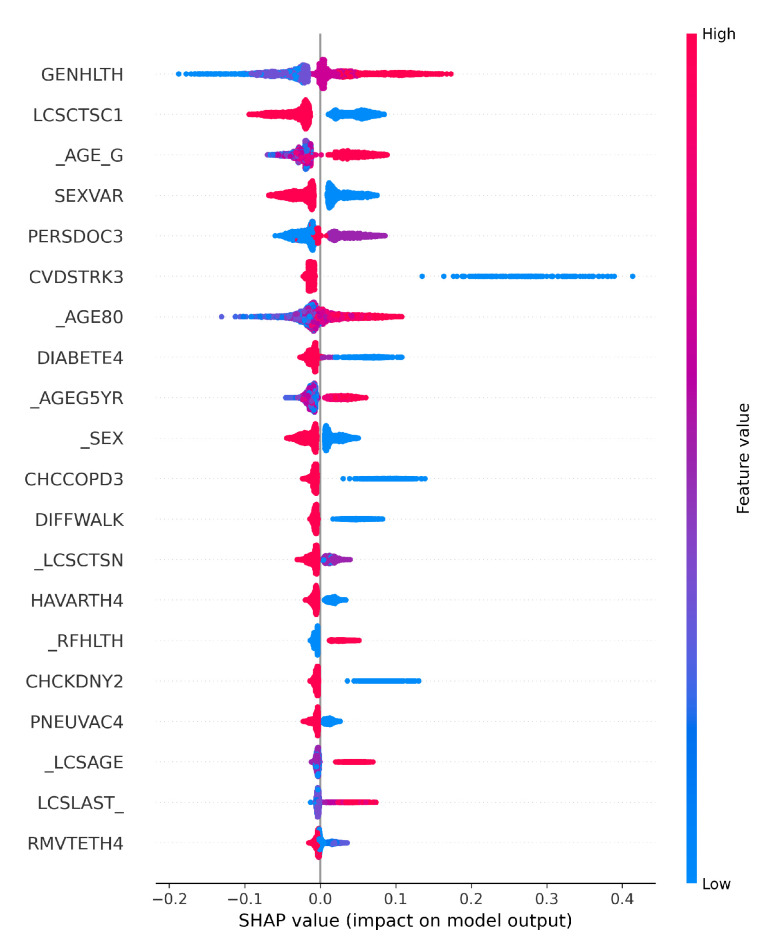
SHAP beeswarm plot illustrating feature-level impact and directionality on the final fused prediction output. Red indicates high feature values, while blue indicates low feature values. Horizontal position reflects contribution toward higher or lower predicted CHD risk.

Figure 14 provides a SHAP beeswarm visualization highlighting feature-level directionality and instance-level variability in contributions to the final fused prediction.

The SHAP beeswarm plot (Figure 14) further reveals directional behavior of the predictors. Poor general health status and a positive history of stroke or diabetes consistently push predictions toward higher CHD risk, while younger age categories tend to contribute negatively to the predicted risk. The distribution of SHAP values highlights instance-level variability, reflecting the adaptive behavior of the gated fusion mechanism rather than rigid global decision rules.

Beyond feature-level attribution, the dual-branch architecture introduces structural interpretability by explicitly separating sensitivity-oriented (recall-focused) and specificity-oriented (precision-focused) decision pathways. This decomposition enables analysis of whether individual predictions are driven more strongly by detection-oriented or verification-oriented reasoning.

Furthermore, the gating mechanism provides additional insight into decision behavior by dynamically weighting the contributions of the two branches at the instance level, allowing for partial tracing of how conflicting objectives are resolved for each sample.

Qualitative evidence from the ablation study further supports this interpretation: removing the veto and rescue rules increases variability in decision outcomes across validation folds, indicating reduced stability. This observation highlights the role of rule-based constraints in enforcing consistent and interpretable decision behavior within the overall architecture.

### 6.10. Joint Interpretation of Statistical Effects and SHAP-Based Feature Importance

To better understand how the proposed model utilizes the available predictors, we compared the univariate statistical group differences with the feature importance scores obtained from the SHAP analysis of the final trained model. Although both analyses examine the relationship between input variables and heart disease status, they reflect fundamentally different analytical perspectives.

The statistical tests quantify marginal differences between the heart disease and non–heart disease groups for each feature independently using contingency analysis and effect-size estimation. In contrast, SHAP values are derived from the fully trained model and capture the multivariate and nonlinear contributions of each feature within the complete prediction pipeline. Consequently, agreement between the two analyses suggests that the model relies on clinically meaningful variables, whereas discrepancies indicate that the model is learning interaction patterns that cannot be detected using univariate statistical analysis alone.

A strong degree of overlap is observed between the two rankings. Several variables with large statistical effect sizes, including _AGEG5YR, _AGE80, GENHLTH, _LCSCTSN, _AGE_G, DIFFWALK, CVDSTRK3, CHCCOPD3, and DIABETE4, also appear among the highest-ranked features in the SHAP analysis. These variables correspond to well-established cardiovascular risk factors, including age, general health condition, mobility limitation, chronic disease history, and metabolic disorders. The agreement between statistical significance and model attribution therefore supports the clinical plausibility of the learned decision patterns.

However, the ranking produced by SHAP is not identical to the ranking obtained from statistical tests. Some variables with strong univariate group differences appear lower in the SHAP importance list, while other variables with moderate statistical effects receive higher importance in the model. This behavior is expected because the proposed Dual-Branch FT-Transformer learns complex nonlinear relationships and feature interactions. When multiple variables encode similar clinical information, the model may rely more heavily on the most informative combination rather than on the variable with the largest individual group difference.

For instance, several age-related variables (_AGEG5YR, _AGE80, and _AGE_G) show strong statistical effects, but their predictive contributions are partially redundant. The attention-based architecture can compress correlated signals and distribute importance across related predictors. Similarly, variables related to lung screening, chronic conditions, and healthcare access demonstrate moderate statistical effects but receive higher SHAP importance because they interact with other predictors within the model.

Overall, this comparison demonstrates that the proposed model preserves the dominant clinical risk patterns present in the dataset while simultaneously capturing higher-order relationships that are not observable through univariate statistical analysis. The consistency between statistical testing and model-based attribution strengthens confidence in both the interpretability and the predictive validity of the proposed approach.

### 6.11. External Cohort Validation on NHANES

To further evaluate the robustness and cross-cohort applicability of the proposed framework, an additional external validation study was conducted using the National Health and Nutrition Examination Survey (NHANES) 2017–March 2020 pre-pandemic dataset [33]. Unlike BRFSS-2024, which primarily contains large-scale behavioral and self-reported surveillance attributes, NHANES provides a substantially different population-health ecosystem that combines demographic information, clinical questionnaires, examination measurements, laboratory biomarkers, metabolic indicators, and lifestyle-related variables. This heterogeneous structure provides a clinically enriched evaluation setting for assessing whether the proposed dual-branch framework remains effective beyond the original BRFSS-2024 cohort.

Multiple NHANES modules were merged using the participant identifier (SEQN), including demographic, blood pressure, diabetes, smoking, physical activity, body measurement, complete blood count, glucose, lipid profile, insulin, inflammation, and medical-condition questionnaire components. The resulting dataset construction and filtering process is summarized in Table 14. After module merging, the initial NHANES dataset contained 15,560 participants and 282 variables. To reduce excessive sparsity while retaining clinically meaningful heterogeneity, variables with more than 70% missingness were removed. Target-construction variables, administrative identifiers, all-NaN variables, and constant variables were then excluded before model development. After target construction and filtering, the final external validation cohort consisted of 9180 participants, with a positive cardiovascular disease prevalence of 9.53%, making it a highly imbalanced dataset.

A binary cardiovascular disease target (CVD_TARGET) was constructed using clinically related cardiovascular-history variables corresponding to congestive heart failure, coronary heart disease, angina, and heart attack. Participants were labeled as positive if any of these cardiovascular conditions were present. To prevent target leakage, all target-construction variables were removed before feature selection and model training. In addition, SEQN was removed as an administrative identifier, and all-NaN and constant variables were excluded. This produced 163 usable candidate features before Phase 1 feature selection, as reported in Table 14.

Importantly, NHANES differs substantially from BRFSS-2024 in both feature composition and data-generation characteristics. While BRFSS primarily relies on large-scale telephone-based behavioral surveillance attributes, NHANES integrates laboratory biomarkers, examination measurements, and clinically oriented health indicators collected under controlled assessment protocols. Consequently, the competitive performance observed on NHANES suggests that the proposed framework can retain balanced predictive behaviour under substantially different feature distributions and clinical variable interactions, rather than relying solely on BRFSS-specific statistical patterns. However, this should be interpreted as evidence of cross-cohort robustness under a leakage-safe re-training protocol rather than direct cross-dataset transfer without adaptation.

The same Phase 1 multi-selector feature fusion strategy used in the BRFSS-2024 experiments was preserved for NHANES. Specifically, Pearson correlation filtering, TabNet mask importance, and HistGradientBoosting permutation importance were jointly applied under a leakage-safe stratified 5-fold protocol. All preprocessing operations, including median imputation, scaling, and feature selection, were fitted only on training folds. This process selected 141 features for Phase 2 modeling, as shown in Table 14. The identical dual-branch FT-Transformer architecture and GateNet-based adaptive fusion strategy were then evaluated against LightGBM, Random Forest, CNN, and simple average fusion.

**Figure 15:**
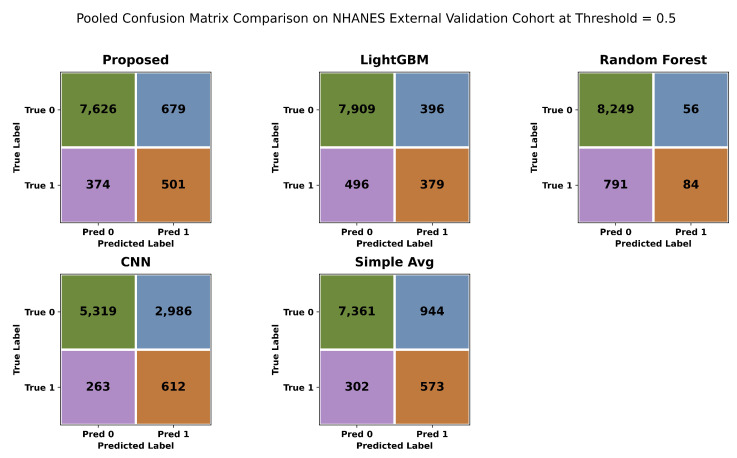
Pooled confusion matrix comparison on the independent NHANES external validation cohort under a unified decision threshold of 0.50. The proposed framework achieves a more balanced error profile compared with LightGBM, Random Forest, CNN, and simple average fusion. Random Forest minimizes false positives but produces a large number of false negatives, whereas CNN achieves high recall at the cost of substantial false-positive escalation. The proposed framework maintains a more balanced trade-off between false positives and false negatives under the independent NHANES cohort.

The external validation results are presented in Table 15, while the pooled confusion matrix comparison is illustrated in Figure 15. The proposed framework achieved the highest overall F1-score (0.4876) and highest AUPRC (0.4677) among all evaluated models while maintaining a balanced precision–recall trade-off under the independent NHANES cohort. Although simple average fusion achieved slightly higher balanced accuracy and recall, it produced substantially more false positives (944) than the proposed framework (679), resulting in a less stable operating profile under imbalanced screening conditions.

The pooled confusion matrices further highlight the different operating behaviors of the comparison models. Random Forest achieved very low false positives but produced a large number of false negatives due to extremely low recall, whereas CNN achieved high recall at the cost of substantial false-positive escalation. In contrast, the proposed framework maintained a more balanced false-positive/false-negative trade-off while preserving the strongest overall F1-score across models.

From a healthcare-screening perspective, these results suggest that the proposed dual-branch gated architecture provides improved stability under severe class imbalance by adaptively regulating precision-oriented and recall-oriented behaviours. Importantly, despite substantial differences in feature composition and data-generation characteristics between NHANES and BRFSS-2024, the proposed framework consistently maintained competitive and balanced operating behaviour across both cohorts, suggesting potential robustness across differing population-health datasets while warranting further external validation on additional cohorts.

### 6.12. Limitations and Directions for Future Work

Despite its strengths, the proposed approach has several limitations. As summarized in Table 16, the increased architectural complexity introduces additional training overhead and greater sensitivity to hyperparameter choices. Future work could explore more parameter-efficient gating mechanisms or partial weight sharing between branches to reduce computational cost while preserving the benefits of architectural decomposition.

Although the proposed framework introduces additional architectural components compared to conventional single-model approaches, the design is intentionally modular. The FT-Transformer backbone accounts for the primary computational cost, while the GateNet component remains lightweight, consisting of a single hidden layer with 16 units. The veto and rescue rules introduce negligible computational overhead, and Phase 1 feature selection is performed offline in a fold-wise manner. Future work will explore parameter-efficient variants and model compression techniques for resource-constrained environments.

Although this study primarily focuses on the BRFSS-2024 dataset, an additional independent cohort evaluation was conducted using NHANES under the same leakage-safe re-training protocol to assess cross-cohort robustness. While this provides encouraging evidence of model stability across substantially different population-health datasets, the evaluation should be interpreted as independent cohort validation with re-training rather than direct cross-dataset transfer without adaptation. Therefore, further validation on additional external, longitudinal, and clinically adjudicated cohorts would strengthen confidence in the broader applicability of the proposed framework. Integrating richer multimodal health data sources, including physiological signals, electronic health records, or wearable sensor measurements, may further improve predictive capability and real-world clinical utility.

Finally, although calibration techniques were applied to improve probability estimates, uncertainty quantification remains an open challenge. Incorporating probabilistic modeling, Bayesian approaches, or confidence-aware decision rules could further enhance reliability and trustworthiness in clinical deployment scenarios.

### 6.13. Broader Implications for Imbalanced Medical ML

Beyond cardiovascular risk prediction, the findings of this study have broader implications for machine learning applications in healthcare. Many medical prediction tasks involve asymmetric error costs and severe class imbalance, where traditional single-objective optimization is often insufficient.

The results suggest that architectural decomposition of competing objectives, combined with adaptive fusion mechanisms, represents a promising direction for future research. Rather than relying solely on increasingly complex loss functions, explicitly modeling competing objectives at the architectural level may provide greater flexibility, interpretability, and performance in imbalanced medical prediction tasks.

## 7. Conclusions

This study presents a leakage-safe, precision-aware deep learning framework for large-scale heart disease risk prediction using the BRFSS-2024 dataset. The proposed Dual-Branch FT-Transformer separates recall-focused detection and precision-focused verification, combining their outputs through a lightweight gating mechanism to better control false negatives and false positives in highly imbalanced health data. A rigorous evaluation pipeline incorporating fold-wise preprocessing, imbalance-aware loss functions, and calibration was used to ensure reliable performance estimates under realistic evaluation conditions. The experimental results demonstrate competitive performance in terms of F1-score, balanced accuracy, and AUPRC relative to strong machine learning and deep learning baselines, while the proposed decision-aware framework provides a more explicitly controlled precision–recall trade-off under severe class imbalance.

Additional evaluation on an independent NHANES cohort under the same leakage-safe re-training protocol further suggested robustness across heterogeneous population-health settings with substantially different feature composition and clinically enriched variables. Although the proposed framework demonstrated competitive and balanced performance across both cohorts, further validation on additional external and longitudinal clinical datasets remains necessary before drawing stronger conclusions regarding real-world clinical deployment.

## Figures and Tables

**Table 2:** Conceptual comparison of representative tabular learning paradigms with the proposed framework in terms of objective formulation, leakage-safe pipeline design, and decision-level error control.

Model	Objective Type	Leakage-Safe Pipeline	Error Trade-Off Control	Calibration Reporting
FT-Transformer	Single objective	Not explicitly addressed	Implicit (threshold tuning)	Rarely reported
TabNet	Single objective	Not explicitly addressed	Implicit (class weighting)	Rarely reported
LightGBM	Single objective	Not explicitly addressed	Threshold-based control	Occasionally reported
CNN-based models	Single objective	Not explicitly addressed	Recall-dominant or implicit	Not reported
**Proposed Framework**	Dual-objective (Recall + Precision)	Explicitly enforced	Explicit (gating + rules)	Evaluated at decision level

**Table 3:** Summary statistics of the BRFSS-2024 modeling dataset (Phase 2 aligned). Missingness refers to the average proportion of missing values across features prior to median imputation.

Attribute	Value
Samples (N)	452,464
Positives (*y* = 1)	42,338
Negatives (*y* = 0)	410,126
Prevalence (%)	9.3572
Imbalance (Neg/Pos)	9.6869
Numeric features (post-cleaning)	283
Selected features (Phase 1 list)	181
Avg. missingness, all numeric (%)	51.7566
Avg. missingness, selected (%)	41.7640

**Table 4:** Baseline models and hyperparameter configurations used for Phase 2 evaluation on all usable BRFSS-2024 features.

Model	Key Hyperparameters
LightGBM	SMOTE applied on training folds only (k=5); $n_{\mathrm{estimators}}=800$, learning rate =0.05, num leaves =64, max depth =−1, min child samples =30, subsample =0.8, column sample by tree =0.8, λ=0, α=0, binary objective, random seed =42
Logistic Regression	StandardScaler (train-only); solver = saga, maximum iterations =5000, class weight = balanced, random seed =42
SVM	StandardScaler (train-only); LinearSVC with class weight = balanced; probability calibration via sigmoid (cv=3); random seed =42
Decision Tree	Max depth = None, minimum samples per leaf =25, class weight = balanced, random seed =42
Random Forest	600 trees, minimum samples per leaf =10, feature sampling = square root, class weight = balanced_subsample, random seed =42
KNN	StandardScaler (train-only); 25 neighbours, distance-weighted voting, Euclidean distance metric
Naive Bayes	StandardScaler (train-only); Gaussian Naive Bayes with default parameters
XGBoost	$n_{\mathrm{estimators}}=1200$, learning rate =0.03, max depth =6, subsample =0.85, column subsample =0.85, λ=1.0, α=0, binary logistic objective, AUPRC evaluation, scale_pos_weight computed per fold
CatBoost	2000 iterations, learning rate =0.03, tree depth =8, Logloss objective, AUC evaluation metric, class weights computed per fold
AE + LR	StandardScaler (train-only); autoencoder: d→256→64→32; AdamW optimisation; latent features classified using Logistic Regression
1D-CNN	StandardScaler (train-only); Conv1D layers (16, 32), kernel size =5, adaptive average pooling, dropout =0.10; BCEWithLogits loss with positive-class weighting
LSTM	StandardScaler (train-only); bidirectional LSTM (hidden size =64), mean pooling, dropout =0.10, BCEWithLogits loss
GRU	StandardScaler (train-only); bidirectional GRU (hidden size =64), mean pooling, dropout =0.10, BCEWithLogits loss

**Symbol definitions:**$n_{\mathrm{estimators}}$ = number of trees/boosting rounds; η = learning rate; num_leaves = maximum number of leaves in LightGBM; *d* = input feature dimensionality; *k* = number of neighbours (KNN) or SMOTE neighbours; λ,α = $\ell_{2}$ and $\ell_{1}$ regularisation coefficients; $N_{\mathrm{neg}},N_{\mathrm{pos}}$ = numbers of negative and positive samples in the training fold; $\mathrm{scale}\_\mathrm{pos}\_\mathrm{weight}=N_{\mathrm{neg}}/N_{\mathrm{pos}}$; $\mathrm{pos}\_\mathrm{weight}=N_{\mathrm{neg}}/N_{\mathrm{pos}}$ in BCEWithLogitsLoss; *h* = hidden-state size in recurrent networks; $k_{s}$ = convolution kernel size; *p* = dropout probability.

**Table 5:** Leakage-safe ablation study of feature representation: comparison between the numeric-only input space and a mixed representation with one-hot encoded categorical variables on BRFSS-2024. The mixed representation substantially increases dimensionality without improving predictive performance, supporting the use of the numeric-only design in the proposed framework.

Representation	No. Features	Precision	Recall	F1	AUROC	AUPRC
Numeric-only	283	0.2465	0.8068	0.3776	0.8541	0.3881
Mixed (One-hot)	40,400	0.2460	0.8067	0.3771	0.8540	0.3874

**Table 6:** Performance comparison of baseline machine learning and deep learning models against the proposed Dual-Branch FT-Transformer on the BRFSS-2024 dataset. Values are reported as mean ± standard deviation over five-fold stratified cross-validation.

Model	Accuracy	Balanced Acc	ROC-AUC	PR-AUC	Precision	Recall	F1
LightGBM	0.91±0.00	0.56±0.00	0.85±0.00	0.38±0.01	0.56±0.01	0.13±0.00	0.21±0.00
Logistic Regression	0.75±0.00	0.77±0.00	0.85±0.00	0.38±0.01	0.25±0.00	0.79±0.00	0.37±0.00
SVM	0.91±0.00	0.55±0.01	0.85±0.00	0.38±0.01	0.57±0.02	0.11±0.02	0.19±0.02
Decision Tree	0.75±0.00	0.71±0.00	0.76±0.00	0.26±0.01	0.22±0.00	0.66±0.01	0.33±0.00
Random Forest	0.85±0.00	0.73±0.00	0.85±0.00	0.37±0.01	0.33±0.00	0.58±0.00	0.42±0.00
KNN	0.91±0.00	0.50±0.00	0.78±0.00	0.28±0.00	0.61±0.05	0.01±0.00	0.02±0.00
Naive Bayes	0.66±0.00	0.72±0.00	0.76±0.00	0.19±0.00	0.19±0.00	0.80±0.00	0.30±0.00
AE + LR	0.74±0.01	0.74±0.01	0.81±0.01	0.29±0.01	0.22±0.01	0.73±0.00	0.34±0.01
1D-CNN	0.74±0.01	0.77±0.00	0.85±0.00	0.38±0.01	0.24±0.01	0.81±0.02	0.37±0.00
LSTM	0.76±0.05	0.76±0.01	0.84±0.00	0.36±0.00	0.25±0.03	0.76±0.08	0.37±0.02
GRU	0.73±0.03	0.76±0.00	0.84±0.00	0.35±0.01	0.23±0.01	0.80±0.04	0.36±0.01
Dual-Branch FT-Transformer (Proposed)	0.86±0.02	0.73±0.02	0.79±0.01	0.38±0.01	0.35±0.03	0.59±0.06	0.43±0.01

**Table 7:** Paired statistical comparison between the proposed dual-branch FT-Transformer and simple average fusion across five cross-validation folds. Mean fold-wise values are reported for F1-score, recall, and precision, together with paired *t*-test and Wilcoxon signed-rank *p*-values, showing that the observed differences are not statistically significant.

Metric	Proposed (Mean)	Avg Fusion (Mean)	*t*-Test (*p*-Value)	Wilcoxon (*p*-Value)
F1-score	0.430	0.431	0.620	0.680
Recall	0.599	0.605	0.480	0.550
Precision	0.338	0.335	0.310	0.420

**Table 8:** Error distribution across three representative models at a fixed decision threshold of 0.50, derived from pooled out-of-fold predictions. The selected models illustrate distinct operating regimes—conservative (LightGBM), recall-dominant (CNN1D), and balanced (proposed model)—to highlight how different architectures influence false-positive and false-negative trade-offs.

Model	False Positives (FP)	False Negatives (FN)	Precision	Recall
LightGBM	4421	36,777	High	Low
CNN1D Dual-Branch	108,803	8011	Low	High
Proposed Model	50,487	16,975	Balanced	Balanced

**Table 9:** Comprehensive error-profile comparison across major baseline models and the proposed framework at a unified decision threshold of 0.50 using pooled out-of-fold predictions. Reporting FP, FN, precision, recall, and F1-score under identical operating conditions enables a consistent and fair comparison of model behavior.

Model	FP	FN	Precision	Recall	F1
Proposed	50,487	16,975	0.334	0.599	0.429
LightGBM	4421	36,777	0.557	0.131	0.212
Random Forest	49,186	17,919	0.332	0.577	0.421
CNN	108,803	8011	0.240	0.811	0.370
Simple Avg	37,835	20,464	0.366	0.517	0.429

**Table 10:** Ablation study of the proposed dual-branch architecture, comparing the recall-only head, precision-only head, simple average fusion, and the final GateNet-based fusion with veto/rescue rules. The results quantify the contribution of branch specialization and decision-level fusion to error control under severe class imbalance.

Variant	F1	Precision	Recall	BalAcc	FP	FN
Recall Head Only	0.378	0.247	0.804	0.775	103,572	8318
Precision Head Only	0.375	0.452	0.320	0.640	16,422	28,780
Simple Average Fusion	0.431	0.335	0.605	0.741	50,966	16,705
GateNet + Veto/Rescue (Final)	0.430	0.349	0.599	0.738	50,487	16,975

**Table 11:** Leakage-safe imputation-strategy ablation under five-fold cross-validation. Comparison of median imputation, mean imputation, and median imputation with missingness indicators shows nearly identical predictive performance, indicating that simple median imputation is sufficient for BRFSS-2024 despite high feature missingness.

Imputation Strategy	F1	Recall	AUPRC	AUROC
Median	0.3776 ± 0.0028	0.8068 ± 0.0028	0.3881 ± 0.0060	0.8541 ± 0.0020
Mean	0.3775 ± 0.0030	0.8067 ± 0.0030	0.3880 ± 0.0060	0.8540 ± 0.0020
Median + Indicators	0.3776 ± 0.0030	0.8068 ± 0.0030	0.3882 ± 0.0060	0.8541 ± 0.0020

**Table 12:** Calibration performance of raw and post-hoc calibrated probabilities for the proposed model. Brier score and expected calibration error (ECE) are reported for raw outputs, Platt scaling, and isotonic regression; lower values indicate better probability calibration.

Method	Brier Score	ECE
Raw	0.0944	0.1027
Platt Scaling	0.0695	0.0040
Isotonic Regression	0.0695	0.0005

**Table 13:** Baseline statistical differences for the top 20 SHAP-ranked features. Adjusted *p*-values were obtained using the Benjamini–Hochberg False Discovery Rate (FDR) procedure. Effect size is reported as Cramér’s *V*. Extremely small adjusted *p*-values are reported as <0.001.

Feature	Test	$p_{\mathrm{FDR}}$	Cramér’s *V*	Magnitude
_AGEG5YR	Chi-square	<0.001	0.2771	Medium
_AGE80	Chi-square	<0.001	0.2478	Medium
GENHLTH	Chi-square	<0.001	0.2410	Medium
_LCSCTSN	Chi-square	<0.001	0.2346	Medium
_AGE_G	Chi-square	<0.001	0.2317	Medium
LCSCTSC1	Chi-square	<0.001	0.2264	Medium
_LCSAGE	Chi-square	<0.001	0.2199	Medium
_RFHLTH	Chi-square	<0.001	0.2095	Medium
DIFFWALK	Chi-square	<0.001	0.2063	Medium
CVDSTRK3	Chi-square	<0.001	0.1998	Medium
CHCCOPD3	Chi-square	<0.001	0.1832	Medium
DIABETE4	Chi-square	<0.001	0.1807	Medium
LCSLAST_	Chi-square	<0.001	0.1776	Medium
HAVARTH4	Chi-square	<0.001	0.1772	Medium
PNEUVAC4	Chi-square	<0.001	0.1653	Medium
CHCKDNY2	Chi-square	<0.001	0.1584	Medium
RMVTETH4	Chi-square	<0.001	0.1548	Medium
PERSDOC3	Chi-square	<0.001	0.1289	Medium
SEXVAR	Chi-square	<0.001	0.0674	Small
_SEX	Chi-square	<0.001	0.0673	Small

**Table 14:** Summary of NHANES external validation dataset construction and preprocessing pipeline.

Property	Value
Merged NHANES participants	15,560
Merged raw variables	282
Missingness threshold	>70% removed
Variables after missingness filtering	173
Target definition variables removed	4
Administrative identifiers removed	1
All-NaN variables removed	1
Constant variables removed	3
Final dataset size	9180
Final feature count before Phase 1 selection	163
Positive class ratio	9.53%
Negative class ratio	90.47%
Phase 1 selected features	141
Validation protocol	Stratified 5-fold CV

**Table 15:** External cohort validation results on the independent NHANES dataset using the same leakage-safe evaluation protocol and Phase 1 selected features. The proposed dual-branch gated framework achieved the highest overall F1-score while maintaining a balanced precision–recall trade-off across major baseline models.

Model	Precision	Recall	BalAcc	F1-Score	AUROC	AUPRC	FP	FN
Proposed	0.4246	0.5726	0.7454	**0.4876**	0.8736	**0.4677**	679	374
LightGBM	0.4890	0.4331	0.6927	0.4594	0.8739	0.4522	396	496
Random Forest	**0.6000**	0.0960	0.5446	0.1655	**0.8794**	0.4285	**56**	791
CNN	0.1701	**0.6994**	0.6699	0.2736	0.7405	0.2271	2986	**263**
Simple Avg	0.3777	0.6549	**0.7716**	0.4791	0.8696	0.4601	944	302

*Note:* Bold values represent the best performance for each evaluation metric.

**Table 16:** Component-wise computational overhead and functional role of the proposed dual-branch framework. The table decomposes the pipeline into feature-selection, backbone modeling, branch prediction, gating, and rule-based control stages to clarify where computational cost is incurred and to show that the additional fusion components introduce only modest overhead.

Component	Execution Stage	Computational Cost	Function
Phase 1 Multi-Selector Feature Selection	Offline (preprocessing)	Moderate (one-time)	Leakage-safe and robust feature construction
FT-Transformer Backbone	Training/Inference	Moderate	Nonlinear representation learning
Dual-Branch Prediction Heads	Training/Inference	Low	Asymmetric objective modeling (recall vs. precision)
GateNet (Hidden Size = 16)	Training/Inference	Very Low	Instance-wise adaptive fusion
Veto and Rescue Rules	Inference only	Negligible	Deterministic error control (FP/FN regulation)

## Data Availability

The BRFSS-2024 dataset used in this study is publicly available from Kaggle at: https://www.kaggle.com/datasets/rudritarahman/cdc-brfss-survey-data-2024 (accessed on 15 September 2025). The dataset is based on the Behavioural Risk Factor Surveillance System (BRFSS), a large-scale health survey conducted by the Centers for Disease Control and Prevention (CDC) to collect information on health-related risk behaviours, chronic conditions, and preventive health practices among adults in the United States. The NHANES 2017–March 2020 pre-pandemic dataset used for the independent cohort evaluation is publicly available from the National Center for Health Statistics (NCHS) at: https://wwwn.cdc.gov/nchs/nhanes/continuousnhanes/default.aspx?BeginYear=2017 (accessed on 13 May 2026). All data analyzed in this study are openly accessible, and no additional restrictions apply. The datasets were used solely for research and analytical purposes in accordance with their respective data usage policies.
